# Composite materials based on covalent organic frameworks for multiple advanced applications

**DOI:** 10.1002/EXP.20220144

**Published:** 2023-05-23

**Authors:** Jia Chen, Yuting Wang, Yongliang Yu, Jianhua Wang, Juewen Liu, Hirotaka Ihara, Hongdeng Qiu

**Affiliations:** ^1^ CAS Key Laboratory of Chemistry of Northwestern Plant Resources and Key Laboratory for Natural Medicine of Gansu Province, Lanzhou Institute of Chemical Physics Chinese Academy of Sciences Lanzhou China; ^2^ Research Center for Analytical Sciences, Department of Chemistry, College of Sciences Northeastern University Shenyang China; ^3^ Department of Chemistry, Waterloo Institute for Nanotechnology University of Waterloo Waterloo Ontario Canada; ^4^ Department of Applied Chemistry and Biochemistry Kumamoto University Chuo‐ku Kumamoto Japan

**Keywords:** advanced applications, COFs‐based composites, covalent organic frameworks

## Abstract

Covalent organic frameworks (COFs) stand for a class of emerging crystalline porous organic materials, which are ingeniously constructed with organic units through strong covalent bonds. Their excellent design capabilities, and uniform and tunable pore structure make them potential materials for various applications. With the continuous development of synthesis technique and nanoscience, COFs have been successfully combined with a variety of functional materials to form COFs‐based composites with superior performance than individual components. This paper offers an overview of the development of different types of COFs‐based composites reported so far, with particular focus on the applications of COFs‐based composites. Moreover, the challenges and future development prospects of COFs‐based composites are presented. We anticipate that the review will provide some inspiration for the further development of COFs‐based composites.

## Graphical abstract

In this review, we have summarized the development of different types of COFs‐based composites reported so far, with particular focus on the representative applications of COFs‐based composites, and the in‐depth insight into their current challenges and future development prospects. We anticipate that this review may provide some inspiration and insights for the further development of COFs‐based composites.

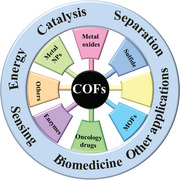

## 1 INTRODUCTION

Covalent organic frameworks (COFs), are a new type of booming crystalline and organic porous materials, which have sprung up in materials science and synthetic chemistry by introducing various organic building units into the frameworks since the first ground breaking work was reported by Yaghi's group.^[^ ^1^ ^]^ COFs have the unique properties of a totally organic metal‐free backbone, well‐defined topology, low mass density, divinable structure, permanent porosity, and exceptional thermal resistance when compared with traditional porous materials and even metal organic frameworks (MOFs).^[^ ^2^, ^3^, ^4^, ^5^ ^]^ To date, a large number of COFs based on various synthetic strategies, such as interface synthesis,^[^ ^6^ ^]^ mechanical synthesis,^[^ ^7^ ^]^ photoacoustic^[^ ^8^ ^]^ and room temperature synthesis^[^ ^9^ ^]^ and different covalent linkages, such as boronic acid bond,^[^ ^1^ ^]^ imine bond,^[^ ^10^ ^]^ triazine bond,^[^ ^9^ ^]^ boronate ester bond,^[^ ^11^ ^]^ hydrazone bond,^[^ ^12^ ^]^ azine bond,^[^ ^13^ ^]^ imide bond,^[^ ^14^ ^]^ carbon‐carbon bond,^[^ ^15^ ^]^ amide bond,^[^ ^16^ ^]^ azo bond^[^ ^17^ ^]^ have been rapidly reported.

**FIGURE 1:**
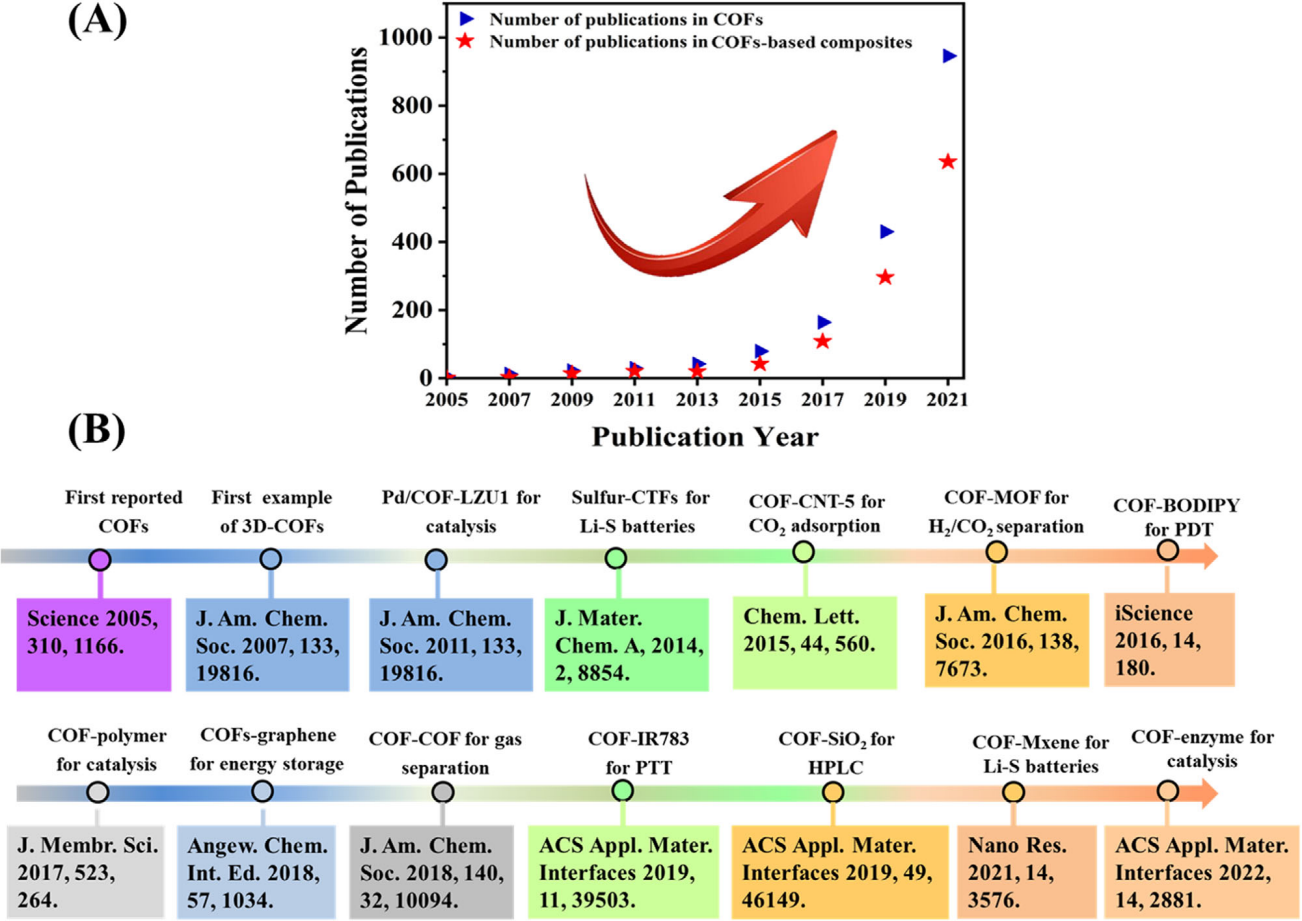
A) Number of publications of covalent organic frameworks (COFs) and COFs‐based composites according to the Web of Science (data obtained 2021). B) Typical examples of COFs‐based composites.

COFs have been seen as fascinating materials used in various fields.^[^ ^18^, ^19^, ^20^, ^21^, ^22^, ^23^ ^]^ However, COFs also Have some weaknesses, including the microcrystalline state of COFs being difficult to dissolve in most solvents, low conductivity, and optical properties, which have limited their further applications. Various materials, including metal nanoparticles (NPs),^[^ ^24^ ^]^ metal oxides materials,^[^ ^25^ ^]^ MOFs,^[^ ^26^, ^27^ ^]^ carbon materials,^[^ ^28^, ^29^, ^30^ ^]^ polymers,^[^ ^31^ ^]^ and others combined with COFs to fabricate COFs‐based composites to possess the advantages of both parent components, and exhibit new unique properties.^[^ ^32^, ^33^, ^34^, ^35^ ^]^ Moreover, COFs‐based composites have mushroomed and attracted extensive attention in many areas. Check out the number of scientific publications in the field of COFs/COFs‐based composites, we can see the published papers in the COFs/COFs‐based composites field have increased significantly in recent years (Figure 1A). Therefore, it is worth summarizing the main application fields of different COFs‐based composites in order to better understand COFs‐based composite. The typical COF‐based composites examples are summarized in Figure 1B.

This review outlined COFs‐based composites that have been successfully fabricated by integrating COFs with other functional materials, including metal NPs, metal oxides materials, carbon materials, sulfides materials, MOFs, enzyme, and so on. In particularly, we focused on the outstanding applications of the composites in catalysis, biomedicine, sensing, energy, separation, etc. Furthermore, at the end of this paper, we summarized the current problems and existential challenges in detail and put forward some possible suggestions.

## 2 TYPES OF COFs‐BASED COMPOSITES

### 2.1 COFs‐metal NPs composites

COFs play a certain limiting effect on the NPs covered on the surface, which has a role similar to that of surfactant and can effectively control the growth of NPs to prevent the aggregation of NPs.^[^ ^36^ ^]^ Among them, the smaller pores in COFs are difficult to accommodate larger size NPs, which is beneficial to improve the selectivity of applications.^[^ ^37^ ^]^ In addition, COFs with some specific functional groups can interact with metal NPs to improve the performance, for example, the π─π bonds of COFs (benzene, C═C, etc.) can improve the efficiency of electron as well as hole transport.^[^ ^38^ ^]^

Catalysis and sensing are the most important applicatiions for COF‐metal NPs, which means that the exploration in other application areas is still in a nascent stage.^[^ ^39^ ^]^ The typical applications of COFs‐metal NPs composites also can be found as listed in Table 1.^[^ ^40^, ^41^, ^42^, ^43^, ^44^, ^45^, ^46^, ^47^, ^48^, ^49^, ^50^, ^51^, ^52^, ^53^ ^]^

**TABLE 1:** Applications of some typical covalent organic framework (COF)‐metal nanoparticles (NPs).

Precursors of COFs	Composites	Application	References
DMTP + TAPB	Au/COF	Reduction of 4‐nitrophenol	[40]
Tp + Pa and Tp + Tta	Ag@TpPa‐1 and Ag@TpTta	Catalytic conversion of CO_2_ and unsaturated amine to carbamate	[41]
Melamine + Et_3_N	Pd@CTF	Catalytic reduction of nitroaromatics	[42]
TAPB + PDA	Fe^0^/TAPB‐PDA COFs	Removal of As (III)	[43]
TFPM + DHBD	AgNPs‐3D‐COF	Reduction of 4‐nitrophenol and degradation of organic dyes	[44]
Tp + Pa	Ru/TpPa‐1	CO_2_ reduction	[45]
Tp + Pa	Ti_3_C_2_/TpPa‐1/Ag	Schottky photocatalytic antibacterial	[46]
PPh_3_‐based trialdehyde + Pa	Pt@Phos‐COF‐1、Pd@Phos‐COF‐1、Au@Phos‐COF‐1、PdAu@Phos‐COF‐1	Catalysis	[47]
Tp + Pa	CdSe/ZnS QD‐grafted COFs	Detection of protein	[48]
TM + TPT	Pt@COF	Oxygen reduction reaction	[49]
TPT + Azo	Au@COF	Detection of Hg^2+^	[50]
Tp + Pa	(Pd/C)@TpPa COFs	Catalysis	[51]
DMTP + TAPB	Au@COFs	Detection of chlorogenic acid	[52]
DAAQ + Tp	DQ‐COF/Ni	Detection and removal of hydrazine	[53]

Abbreviations: Azo, 4, 4′‐azodianiline; BD, benzidine; DAAQ, 2, 6‐diaminoanthraquinone; DHBD, 3,3′‐dihydroxybenzidine; DMTP, 2,5‐dimethoxyterephthaldehyde; Pa, *p*‐phenylenediamine; PDA, terephthalaldehyde; PPh_3_, triphenylphosphine; TAPB, 1,3,5‐tris(4‐aminophenyl)benzene; TFPM, tetra‐(4‐formylphenyl)methane; TM, 2,4,6‐trimethyl‐1,3,5‐trizaine; Tp, 1,3,5‐triformylphloroglucinol; TPT, 1,3,5‐tris‐(4‐formylphenyl)triazine; Tta, 4,4′,4′‐(1,3,5‐Triazine‐2,4,6‐triyl)trianiline.

#### 2.1.1 Application of COFs‐metal NPs composites in catalysis

COFs have heterogeneity in which the ordered framework facilitates charge carrier transport and the open porous structure facilitates mass transport, while their insolubility as well as stability allow for recycling.^[^ ^54^ ^]^

**FIGURE 2:**
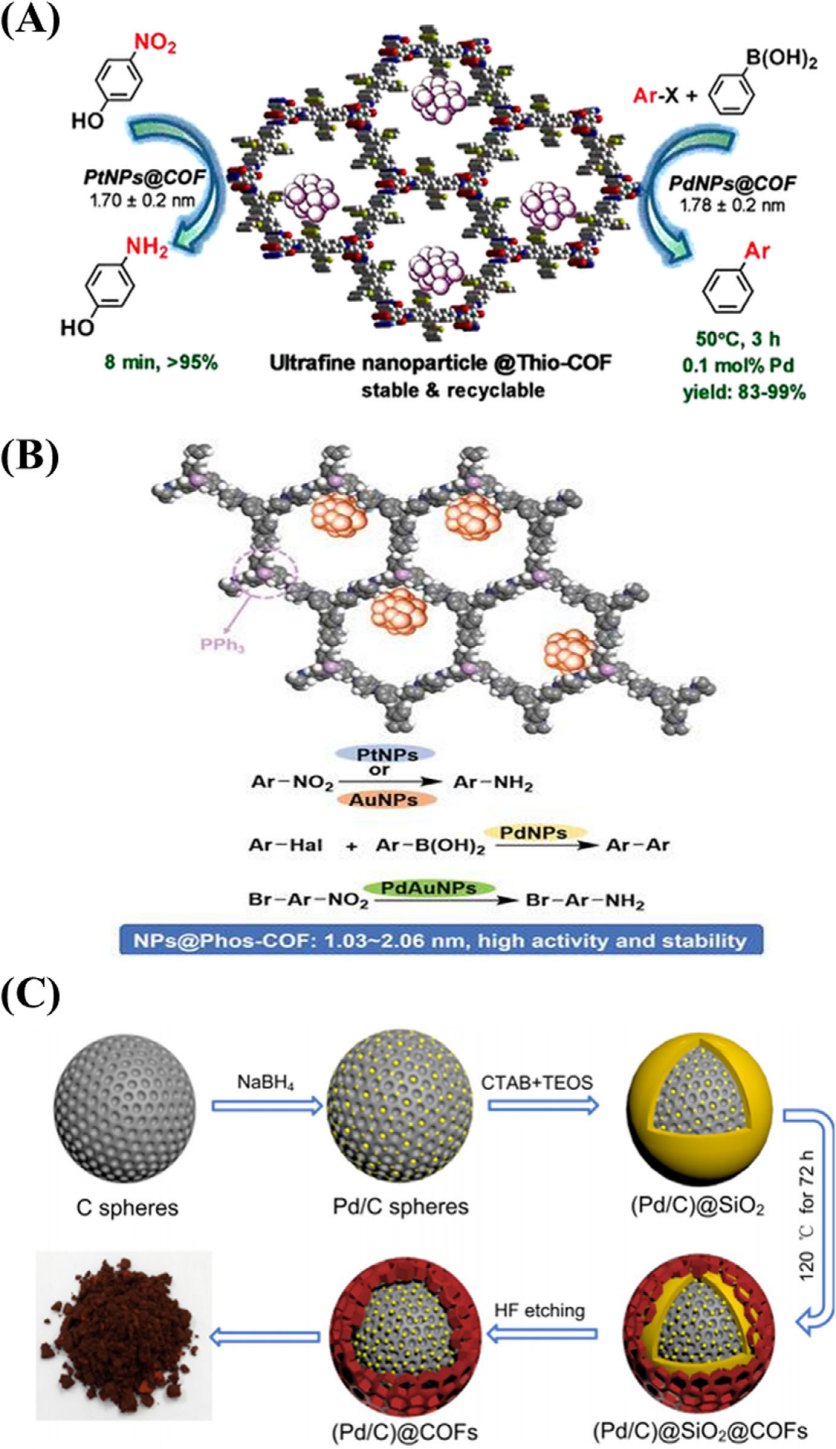
A) Pd nanoparticles (NPs)/Pt NPs confined in thioether‐containing covalent organic frameworks (COFs) and their catalytic applications. Adapted with permission.^[^ ^24^ ^]^ Copyright 2017, American Chemical Society. B) Broad‐scope NPs@phos‐COFs and their multiple catalytic reactions. Adapted with permission.^[^ ^47^ ^]^ Copyright 2020, WILEY‐VCH. C) Synthesis of (Pd/C)@TpPa COFs. Adapted with permission.^[^ ^51^ ^]^ Copyright 2021, Elsevier.

Pd/COF‐LZU1 was first fabricated in 2011 and proved to have excellent catalytic activity.^[^ ^55^ ^]^ This work promoted the catalytic application of functional COFs to a certain extent. In 2014, Banerjee's group reported the incorporation of Pd(II) complex and Pd(0) nanoparticles on TpPa‐1 COFs.^[^ ^56^ ^]^ Impressively, the hybrids were suitable for heterogeneous catalysis under both highly basic and acidic conditions. However, the particle size of Pd NPs was much larger than the pore size of the COFs. The deposition of metal NPs on COFs surface increased the possibility of leaching of NPs in the catalytic cycling reaction and affected the reusability of the catalyst. In order to address the problem, carrier materials with strong interactions with Pd NPs, especially heteroatom‐doped materials, such as quaternary ammonium (QA) salt‐decorated COFs, were used to stabilize Pd NPs to prepare Pd@COF‐QA hybrid materials.^[^ ^57^ ^]^ Meanwhile, the average size (2.4 nm) of Pd NPs was smaller than the pore size (3.8 nm) of COF‐QA, demonstrating that Pd NPs could be deposited in the pores. A series of experimental results showed that the prepared Pd@COF‐QA material could be acted as an excellent phase transfer catalyst to improve aqueous Suzuki‐Miyaura coupling reaction under mild reaction conditions. In 2017, thioether‐containing COFs, have acted as catalyst supports to immobilize metallic Pt NPs and Pd NPs. Both Pd NPs and Pt NPs immobilized within the COFs cavity have narrow size (1.7 ± 0.2 nm) and high loading levels. A series of experiments show that the crystallinity of COFs as well as thioether group in the cavity play an important role in the controlled synthesis of metallic NPs. Meanwhile, it is found that the prepared material is an excellent asymmetric heterogeneous catalyst that effectively promotes Henry and reductive Heck reactions with strong stereoselectivity and high yield (Figure 2A). In addition, this catalyst exhibited good recyclability and stability.^[^ ^24^ ^]^ It is known that triphenyl phosphine has a strong binding affinity for various metal ions and is one of the most commonly used ligands in catalysts. Thus, triphenylphosphine‐based COF (Phos‐COF‐1) was employed as a solid support for the confined growth of ultrafine mono‐ and bimetallic nanoparticles (Pt NPs, Pd NPs, Au NPs, and PdAu NPs), it is demonstrated that the prepared Phos‐COF‐1‐supported NPs display high catalytic activities, good stability, and recyclability toward multiple coupling and reduction reaction (Figure 2B).^[^ ^47^ ^]^

In addition, a protective layer can be designed to overcome the etching of metal NPs. For example, a mesoporous ceria shell was introduced to hinder the Pd NPs leaching and block the Pd NPs aggregation.^[^ ^58^ ^]^ In 2021, Zhang et al. encapsulated Pd NPs into hollow spherical TpPa COFs and prepared yolk–shell (Pd/C)@TpPa COFs using a template method (Figure 2C).^[^ ^51^ ^]^ In this work, TpPa COFs, acted as a protective layer to hinder the Pd NPs leaching in heterogeneous catalysis and had excellent molecular‐size selectivity. Under the optimized experimental conditions, (Pd/C)@TpPa COFs achieved a high conversion at 0.05 mol% Pd loading and the size cutoff efficiency for aryl benzene amounting to 100%. Impressively, the catalyst showed good recyclability.

Exploration of COFs‐metal NPs catalysts involves various catalysis aspects. For example, NPs@COFs composites can also catalyze the oxygen reduction reaction,^[^ ^59^ ^]^ oxygen evolution reaction (OER),^[^ ^60^ ^]^ as well as hydrogen evolution reaction (HER).^[^ ^61^ ^]^ Most reactions are highly efficient (80% to 99% yield) within 2 h.

#### 2.1.2 Application of COFs‐metal NPs composites in sensing

The development of nanoscience has driven innovation of all kinds of sensors by exploiting nanomaterials inimitable chemical and physical properties.^[^ ^62^, ^63^, ^64^ ^]^ COF‐metal NPs are endowed with unparalleled sensing properties due to their various constituent and collaborative functions.^[^ ^65^ ^]^

**FIGURE 3:**
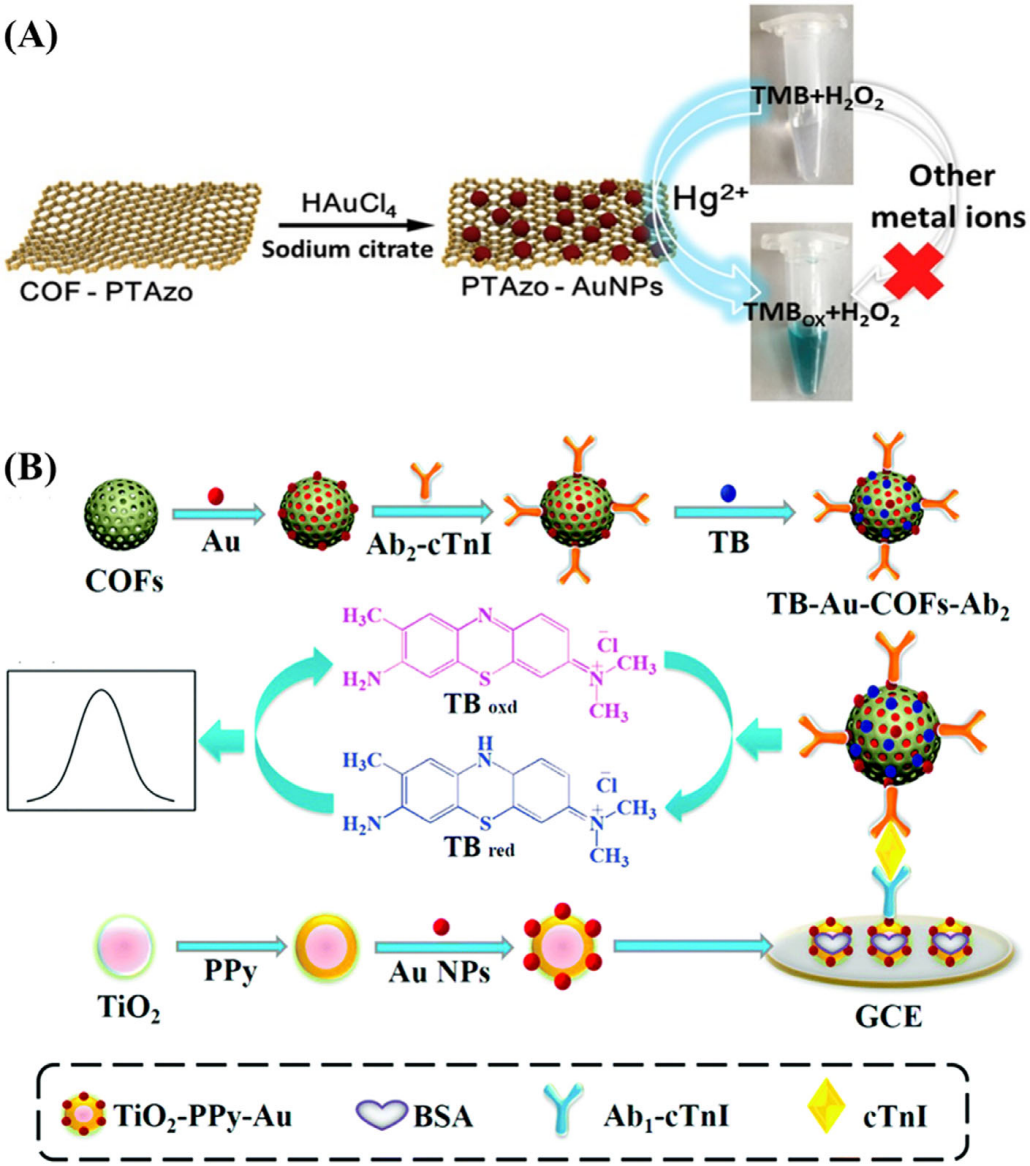
A) The construction of COF‐Au nanoparticles (NPs) for colorimetric detection of Hg^2+^. Adapted with permission.^[^ ^69^ ^]^ Copyright 2019, Elsevier. B) The preparation of the TB‐Au‐COFs‐Ab_2_ labels and the construction of the sandwich‐type electrochemical immunosensor. Adapted with permission.^[^ ^70^ ^]^ Copyright 2018, Elsevier. COF, covalent organic framework.

Colorimetric sensing has many unique advantages, such as being intuitive, and visible to the naked eye.^[^ ^66^, ^67^, ^68^ ^]^ In situ growth of Au NPs on COFs was reported as colorimetric sensors.^[^ ^69^ ^]^ Based on the stable peroxidase‐mimicking activity, when Hg(II) and H_2_O_2_ were present, the color of 3,3,5,5‐tetramethylbenzidine (TMB) changed from colorless to blue, and the color produced by TMB was proportional to the Hg(II) concentration (Figure 3A). Moreover, the system exhibited high selectivity due to the strong affinity between Au and Hg.

At present, progress in developing electrochemical sensing based on COFs has been successfully established to detect environmental pollutants and biomedical analysis. An interesting example is the preparation of a novel sandwich electrochemical sensor from a composites of troponin.^[^ ^70^ ^]^ To facilitate electron transfer, Au NPs were served to modify TiO_2_‐PPy by Au─NH_2_ bonds, which loaded more primary antibodies (Ab_1_). The electronic medium toluidine blue as well as Au NPs‐doped COF were used to achieve signal amplification. TB‐Au‐COF offered more active sites for fixing antibodies (Ab_2_). Under the best conditions, the limit of detection (LOD) of the designed electrochemical immunosensor was as low as 0.17 pg mL^−1^ (Figure 3B). In addition, TAPB‐DMTP COF/Au NPs composites were constructed by the solution infiltration method, and then used to build an advanced electrochemical sensor for chlorogenic acid detection.^[^ ^52^ ^]^ The electrochemical sensor had a wide linear range (1.0 × 10^−8^∼4.0 × 10^−5^ mol L^−1^) as well as a low LOD (9.5 × 10^−9^ mol L^−1^). After 100 cycles, the sensor still maintained extreme stability and catalytic activity.

Based on the presence of ample anthraquinone sites as well as the high surface area, DAAQ‐TFP COF (DQ‐COF) exhibited a hydrazine removal capacity up to 1108 mg g^−1^.^[^ ^53^ ^]^ The detection of hydrazine in this material was further realized by amperometric technology. Compared with other hydrazine sensors (such as Fe_3_O_4_ and NiCo_2_O_4_),^[^ ^71^, ^72^ ^]^ DQ‐COF/Ni has a lower LOD (0.07 μM). The performance is far superior to that of existing porous materials, which is attributable to the synergy of Ni matrix‐enhanced electron transfer and electroactive DQ‐COF.

In addition to hydrazine, other targets such as dopamine, catechol, hydroquinone, and resorcinol have also been detection using COFs‐metal NPs electrochemical sensors.^[^ ^73^, ^74^ ^]^

#### 2.1.3 Application of COFs‐metal NPs composites in biomedicine

Through continuous efforts, COFs‐metal NPs composites have been used to achieve targeted delivery of glucose oxidase,^[^ ^75^ ^]^ photodynamic and photothermal therapy,^[^ ^76^, ^77^ ^]^ pH‐responsice chemo/photothermal/chemodynamic synergistic therapy for cancer.^[^ ^78^ ^]^

The combination of photodynamic therapy (PDT) as well as photothermal therapy (PTT) has broad prospects in the treatment of tumors. In order to realize a satisfactory anti‐tumor effect, selecting an appropriate photosensitizer is a prerequisite. Hu et al. used CuSe as the ideal photothermal agent combine with photosensitizer COF to construct a bifunctional tumor therapeutic agent.^[^ ^77^ ^]^ The photodynamic effect is significantly enhanced after the coordination of COF with CuSe NPs. Meanwhile, the obtained COF‐CuSe nanocomposite exhibits satisfactory PTT effect, and the photothermal conversion efficiency reaches 26.34%. Both in vivo and in vitro experiment data demonstrate that the COF‐CuSe nanoplatform is an ideal platform for PTT and PDT.

A facile room‐temperature synthesis method was used to produce flower‐like COFs with controllable shapes.^[^ ^79^ ^]^ In addition, flower‐like hollow COFs loaded with Au NPs were successfully used for high‐efficiency, selective capture of ultratrace brain natriuretic peptide from human serum samples.^[^ ^39^ ^]^ This means that COFs‐Au NPs can act as a fascinating enrichment probe in biological as well as clinical analysis.

Yuan et al. constructed a pH‐responsive COFs‐based organic/inorganic hybrid platform.^[^ ^80^ ^]^ In the experiment, the zinc porphyrin COF (ZnCOF) was synthesized using MnO_2_ as a template, and then bovine serum albumin was selected as the reducing agent and stabilizer to adsorb on the MnO_2_/ZnCOF surface for the in situ preparation of Au NPs. The produced MnO_2_/ZnCOF@Au&BSA nanosystem could act as an effective nano‐therapeutic agent for FL‐enhanced tumor imaging and cancer therapy.

### 2.2 COFs‐metal oxides composites

COFs‐metal oxides composites are one of the most reported COFs‐based composites.^[^ ^81^ ^]^ COF‐metal oxide composites have made significant progress in catalysis,^[^ ^82^ ^]^ adsorption,^[^ ^83^ ^]^ PTT,^[^ ^84^ ^]^ lithium‐sulfur batteries,^[^ ^85^ ^]^ gas separation,^[^ ^86^ ^]^ absorption,^[^ ^87^, ^88^ ^]^ and sensor fields.^[^ ^89^ ^]^

#### 2.2.1 Application of COFs‐metal oxides composites in catalysis

Due to the excellent stability of COFs in most solvents, catalysts designed and synthesized on the basis of COFs can be easily recycled from different catalytic systems by centrifugation and drying steps.^[^ ^90^, ^91^, ^92^, ^93^ ^]^

**FIGURE 4:**
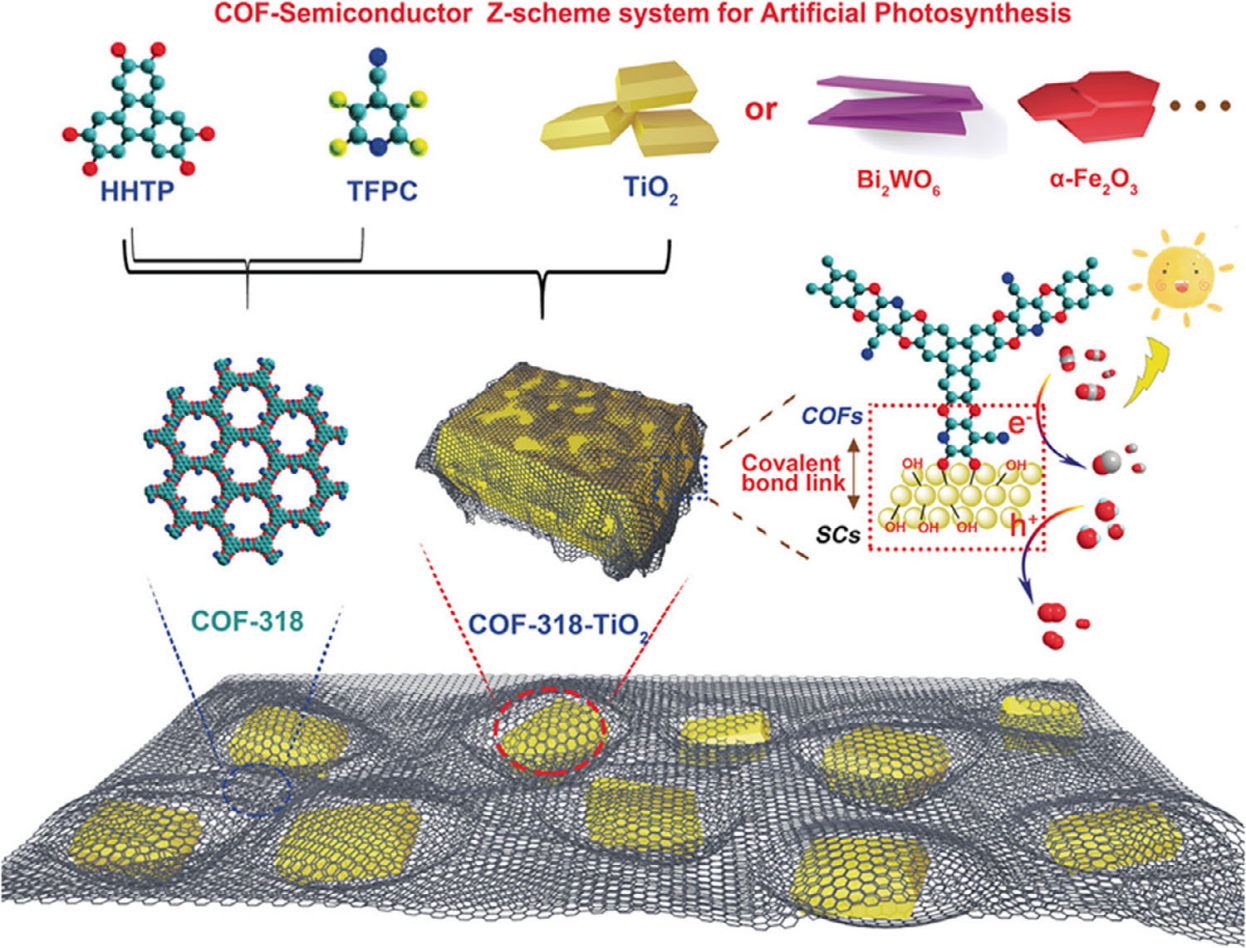
Schematic illustration of covalently linked various semiconductor‐COF Z‐Scheme photocatalysts. Adapted with permission.^[^ ^96^ ^]^ Copyright 2020, American Chemical Society. COF, covalent organic framework.

Zhang et al. reported that crystallized COFs were used as supports to grow Fe^3+^‐doped TiO_2_ NPs with an average particle size of 2.3 nm.^[^ ^82^ ^]^ The synthesized Fe‐TiO_2_@COF demonstrated excellent photocatalytic activity under ambient light and could degrade methylene blue. Among various types of COFs‐metal oxides composites, how to integrate COFs and metal oxides inorganic tightly is still a huge challenge. In another work, COFs and inorganic semiconductor material TiO_2_ were designed to form Z‐scheme hybrid (TiO_2_‐TpPa‐1‐COF) for the first time.^[^ ^94^ ^]^ Covalent bonds can act as a bridge in the heterostructure to enhance the catalytic performance by separating the electron‐hole and transferring photogenerated electrons in the photocatalytic process.^[^ ^95^ ^]^ Lan et al. constructed COF‐semiconductor Z‐type photocatalysts by integrating a variety of semiconductors (α‐Fe_2_O_3_, TiO_2_, and Bi_2_WO_6_) with COFs (COF‐316/318) and prepared different COF Z‐scheme semiconductor catalysts (Figure 4).^[^ ^96^ ^]^ The prepared COF‐318‐TiO_2_ Z‐scheme heterojunction reveals better catalytic performance than that of the physical‐mixture composites. Through density functional theory calculations and a series of characterizations, the authors demonstrated that effective covalent coupling between COFs and semiconductors enabled efficient transfer of light‐generated electrons between organic functional groups and semiconductors.

#### 2.2.2 Application of COFs‐metal oxide composites in extraction

Adsorption and separation have recently attracted more and more concern. The pre‐designed pores of COFs are conducive to the adsorption and separation of various substances: including biomarkers (hormone, protein),^[^ ^97^ ^]^ aromatic compounds,^[^ ^98^ ^]^ pollutants,^[^ ^99^ ^]^ and heavy metal ions.^[^ ^100^ ^]^

The first example of the use of magnetic triazine‐based COFs for magnetic separation was reported in 2011, subsequently, the field is growing rapidly.^[^ ^83^ ^]^ Table 2 lists the representative applications of COFs‐metal oxides in magnetic solid‐phase extraction (MSPE).^[^ ^101^, ^102^, ^103^, ^104^, ^105^, ^106^, ^107^, ^108^, ^109^, ^110^, ^111^ ^]^

**TABLE 2:** Application and analytical performance of typical magnetic covalent organic frameworks (COFs) in magnetic solid phase extraction.

Extraction material	Precursors of COFs	Sorbent dosage	Analytes	Real Samples	Extraction time (min)	LOD	Recovery	Ref.
Fe_3_O_4_@COF	Tp + TAM	0.4 g L^−1^	Bisphenol A	Milk, river water	10	4 pg mL^−1^	93.8 ± 1.4%– 107.3 ± 1.2%	[101]
Fe_3_O_4_@TpBD	Tp + BD	10 mg	Disinfection byproducts	Laboratory tap water, swimming pool water	7	0.07–1.81 ng L^−1^	75.5–118.5%	[102]
Fe_3_O_4_@TPPCl_4_	Tp + PCl_4_	0.8 mg	Polychlorinated naphthalenes	Fine particulate matter	15	0.005–0.325 ng L^−1^	93.11–105.81%	[103]
Fe_3_O_4_@COF@Zr^4+^	BD + Tp	25 mg	Organophosphorus pesticides	Vegetables	30	0.7–3.0 μg kg^−1^	87–121%	[104]
MCNT@COFs	TAPB + TFTA	15 mg	Polybrominated diphenyl ethers	Wastewater, tap water, bottled purified water	20	0.0045–0.018 ng L^−1^	> 80%	[105]
Fe_3_O_4_@COF‐COOH	Pa + DAA + TP	10 mg	Organic pollutants	Lake water	10	(Polycyclic aromatic hydrocarbons) 0.003–0.008 μg L^−1^, (tetracyclines) 0.02–0.06 μg L^−1^, (triphenylmethane dyes) 0.006–0.008 μg L^−1^	93.6–105.8%	[106]
Fe_3_O_4_@COFs	TAPB + DVP	6 mg	Polycyclic aromatic hydrocarbons	Food	9	1.84–8.35 ng kg^−1^	83.2–119.3%	[107]
Fe_3_O_4_@A‐TpBD@NH‐MIL‐125 (Ti)	Tp + BD	15 mg	Endocrine–disrupting chemicals	Milk	15	0.37–0.85 μg L^−1^	92.1–118.3%	[108]
Fe_3_O_4_@SiO_2_@COF‐V	TAPB + Dva	20 mg	Polybrominated diphenyl ethers	Spring water, tap water, lake water, drinking water	15	0.12–0.38 ng L^−1^	79.2–106.6%	[109]
Fe_3_O_4_@TbBD‐COOH	Tb + BD	15 mg	Sulfonamides	Beef, chicken, and pork samples	40	0.1–0.4 μg kg^−1^	85.34–102.61%	[110]
Fe_3_O_4_@TbBD	Tb + BD	20 mg	Estrogens	Urine samples	30	0.2–4.7 ng L^−1^	80.6–111.6%	[111]

Abbreviations: DAA, 2,5‐diaminobenzoic acid; DCB, 1,4‐dicyanobenzene; Dva, 2,5‐divinylter‐ephthalaldehyde; DVP, 2,5‐divinylterephthalaldehyde; PCl_4_, 2,2′,5,5′‐tetrachlorobenzidine; TAM, tetra(p‐aminophenyl)‐methane); Tb, 1,3,5‐triformylbenzene; TFTA, 2,3,5,6‐tetrafluoro‐p‐benzaldehyde.

In the above examples, most of them make use of Fe_3_O_4_ as the carrier to build a magnetic COFs for MSPE. For example, Fe_3_O_4_@TbBd nanobeads as a new MSPE extractant can selectively isolate and enrich trace amounts of estrogens from human urine samples. High enrichment factors (75−197 fold) as well as wide linearity range (0.005–10 g L^−1^) were observed using MSPE and high performance liquid chromatography‐mass spectrometer (HPLC‐MS).^[^ ^111^ ^]^

**FIGURE 5:**
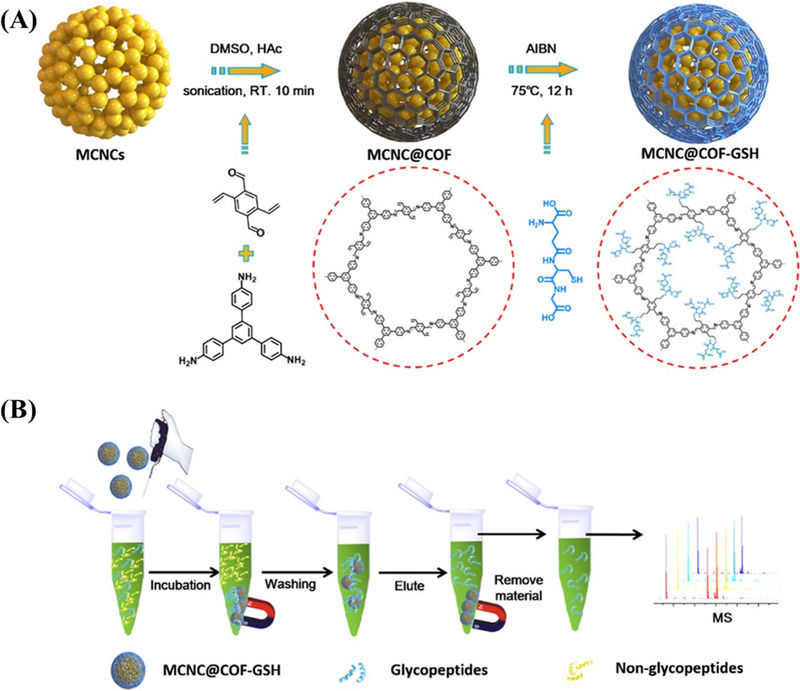
A) Schematic illustration for the synthesis of the MCNC@COF‐GSH. B) Workflow of N‐linked glycopeptide enrichment. Adapted with permission.^[^ ^112^ ^]^ Copyright 2019, American Chemical Society. COF, covalent organic framework.

Luo et al. synthesized glutathione (GSH) ‐functionalized magnetic COFs microspheres by using magnetic crystal nanoclusters as the core, COFs as the shell, and GSH with good biocompatibility and high hydrophilicity as the surfactant.^[^ ^112^ ^]^ As expected to design, GSH‐functionalized magnetic COFs showed excellent size exclusion effect, good selectivity, excellent sensitivity, and excellent enrichment performance for endogenous N‐linked glycopeptide (Figure 5). Surprisingly, a total of 143 endogenous N‐linked glycopeptide species were identified from 10 μL human saliva samples treated with GSH‐functionalized magnetic COFs, showing an unprecedented high‐enrichment efficiency.

The synthesis method of low‐cost COFs‐based composites is beneficial to further promote the practical application of COFs‐based composites. Zhao et al. prepared a new Fe_3_O_4_@[NH_2_]‐COF using a room temperature synthesis strategy. The prepared magnetic COFs showed outstanding extraction performance for nine types of perfluoroalkyl acids (PFAAs) due to the strong electrostatic interaction as well as hydrophobic interaction.^[^ ^113^ ^]^ The linearity of the six analytes was from 10 to 1000 ng L^−1^, and the LODs were between 0.05 and 0.38 ng L^−1^ by HPLC‐MS/MS. The applicability of this strategy was further studied by analyzing nine kinds of PFAAs in rain water, pond water, tap water, and waste water samples. The recoveries of 9 PFAAs were 72.1–113.3%, 74.7–115.1%, 74.8–115.4%, 78.5–113.7%, respectively. Cai et al. established a bouquet‐shaped magnetic TpPa‐1 and used it as an adsorbent for MSPE trace polycyclic aromatic hydrocarbons (PAHs) in environmental samples.^[^ ^114^ ^]^ Quantitative extraction of six analysts (10 ng) required only 5 mg magnetic TpPa‐1, and the recovery rate of most PAHs was above 90%, which showed a high extraction efficiency and excellent enhancement performance of magnetic TpPa‐1.

Since traditional Fe_3_O_4_ is prone to oxidation, agglomeration, and instability in acidic media. NiFe_2_O_4_, a heterotrophic photo‐Fenton catalyst for the degradation of organic pollutants, has arose much concern due to its outstanding chemical stability as well as magnetic properties.^[^ ^115^, ^116^ ^]^ Herein, NiFe_2_O_4_ can be considered as one of the excellent substitutes for traditional magnetic Fe_3_O_4_ NPs.^[^ ^117^ ^]^ NiFe_2_O_4_@COFs nanocomposites were prepared by rapid room‐temperature synthesis. Owing to π–π, hydrophobic interactions as well as van der Waals forces, the adsorption capacity of NiFe_2_O_4_@COFs for brominated flame retardants was stronger than that of carbon nanotube materials. Under the optimal experimental conditions, NiFe_2_O_4_@COFs showed excellent adsorption properties with a low LOD (0.03–1.9 μg L^−1^), and a wide linear range (0.11–1000 μg L^−1^). In addition, the obtained magnetic material acted as an extractant to determine five brominated flame retardants in real samples, and the recovery rate was between 91.5% and 102%.

#### 2.2.3 Application of COFs‐metal oxides composites in biomedicine

PTT to ablate cancer cells by targeting radiation photosensitizer molecules to emit heat has been shown to be an effective approach to cancer treatment.^[^ ^118^ ^]^ The open structure of the conjugated microporous polymer is conducive to heat conduction.^[^ ^119^ ^]^ Hence, the use of COFs can be foreseen as a novel and attractive avenue for the development of organic photothermal agents.^[^ ^120^ ^]^ There have been some designs based on COFs‐metal oxides to achieve PTT for cancer cells.^[^ ^80^, ^84^ ^]^ Wang's group further investigated the potential of Fe_3_O_4_@COF for PTT.^[^ ^84^ ^]^ In the work, a core–shell type Fe_3_O_4_@COF (TpBD) was synthesized using Fe_3_O_4_ as a template and modified with polyethylene glycol (PEG) in order to increase the stability of the material. The characterization data manifested that the pore size of TpBD was 1.3–2.0 nm, the specific surface area was 1346 m^2^ g^−1^, and the thickness of shell was 100 nm. The photothermal experiment displayed that the photothermal conversion efficiency of Fe_3_O_4_@COF‐100 was 21.5% at 785 nm, which was 2–3 times higher than that of Fe_3_O_4_ nanoclusters alone.

**FIGURE 6:**
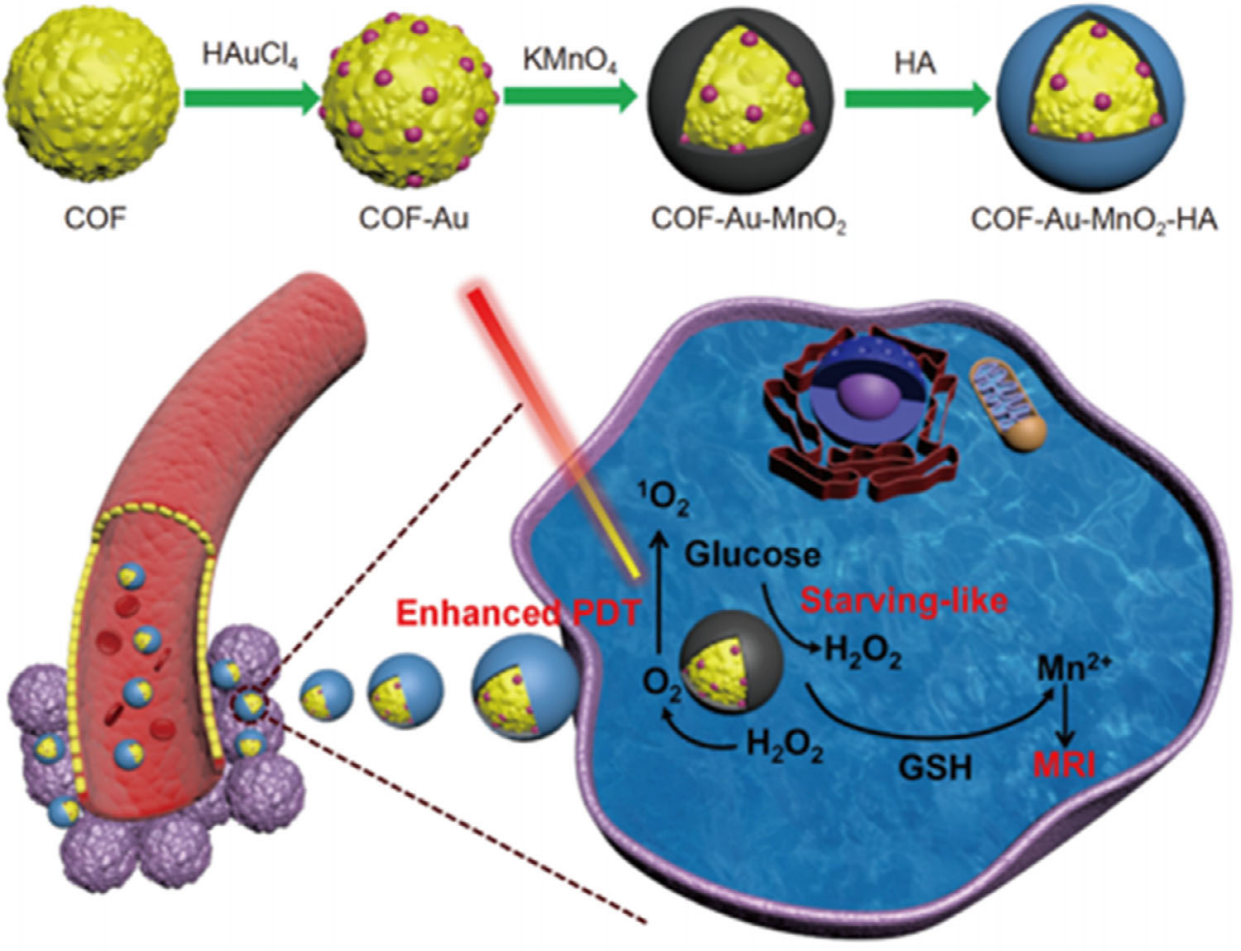
Schematic illustration of the synthetic process of COF‐Au‐MnO_2_‐HA and the therapeutic mechanism. Adapted with permission.^[^ ^123^ ^]^ Copyright 2021, Elsevier. COF, covalent organic framework.

Another effective antitumor approach is PDT, which utilizes the combination of three kinds of non‐toxic components, light source, photosensitizers, as well as tissue oxygen, to generate reactive oxygen species (ROS) that result in oxidative damage to cellular components such as DNA, lipids, as well as proteins.^[^ ^121^, ^122^ ^]^ That is, after the photosensitizer absorbs light energy, PDT kills cancer cells by exciting oxygen (O_2_) into ROS. Due to the limitation of the hypoxic microenvironment in tumor cells, many photodynamic therapies have certain limitations, and it is difficult to generate highly cytotoxic ROS against tumors. Here, COF was selected as a platform to combine with Au NPs, and then a layer of MnO_2_ was coated on this composite, finally introducing hyaluronic acid to increase biocompatibility (Figure 6).^[^ ^123^ ^]^ The formed multifunctional composites displayed good cancer cell killing ability and anti‐tumor effect via catalytic cascade reactions.

### 2.3 COFs‐carbon composites

Carbon material including graphene, carbon nanotubes (CNTs), graphene oxide (GO), carbon dots (CDs), and other categories with outstanding physical and chemical characteristics combined with COFs are used for supercapacitor,^[^ ^124^ ^]^ rechargeable batteries,^[^ ^125^ ^]^ biomedicine,^[^ ^126^ ^]^ and so on.^[^ ^127^, ^128^, ^129^, ^130^, ^131^, ^132^, ^133^ ^]^

#### 2.3.1 Applications of COFs‐carbon composites in energy

A vital energy storage device is a supercapacitors (SCs), which is an electrochemical double‐layer capacitor based on electrode materials.^[^ ^129^ ^]^ Although multiple high‐rate charge carrier transport pathways are realized in COFs, the conductivity of COFs is limited by the presence of disordered regions as well as particle boundaries.^[^ ^134^, ^135^, ^136^ ^]^ Thus, pristine COFs are usually grown on or mixed with conductive materials to obtain SCs.^[^ ^137^, ^138^, ^139^ ^]^

Sun et al. prepared COFs/GO hybrid materials and tested its energy storage performance.^[^ ^28^ ^]^ During the preparation process, benzene‐1,4‐diboronic acid was first covalently grafted onto GO, and then served as the nucleation site for COF‐1 growth to obtain COFs composites growing perpendicular to the substrate. It is worth mentioning that the carbonized composites can still maintain its unique 3D structure and exhibit excellent electrochemical performance in the supercapacitor test.

**FIGURE 7:**
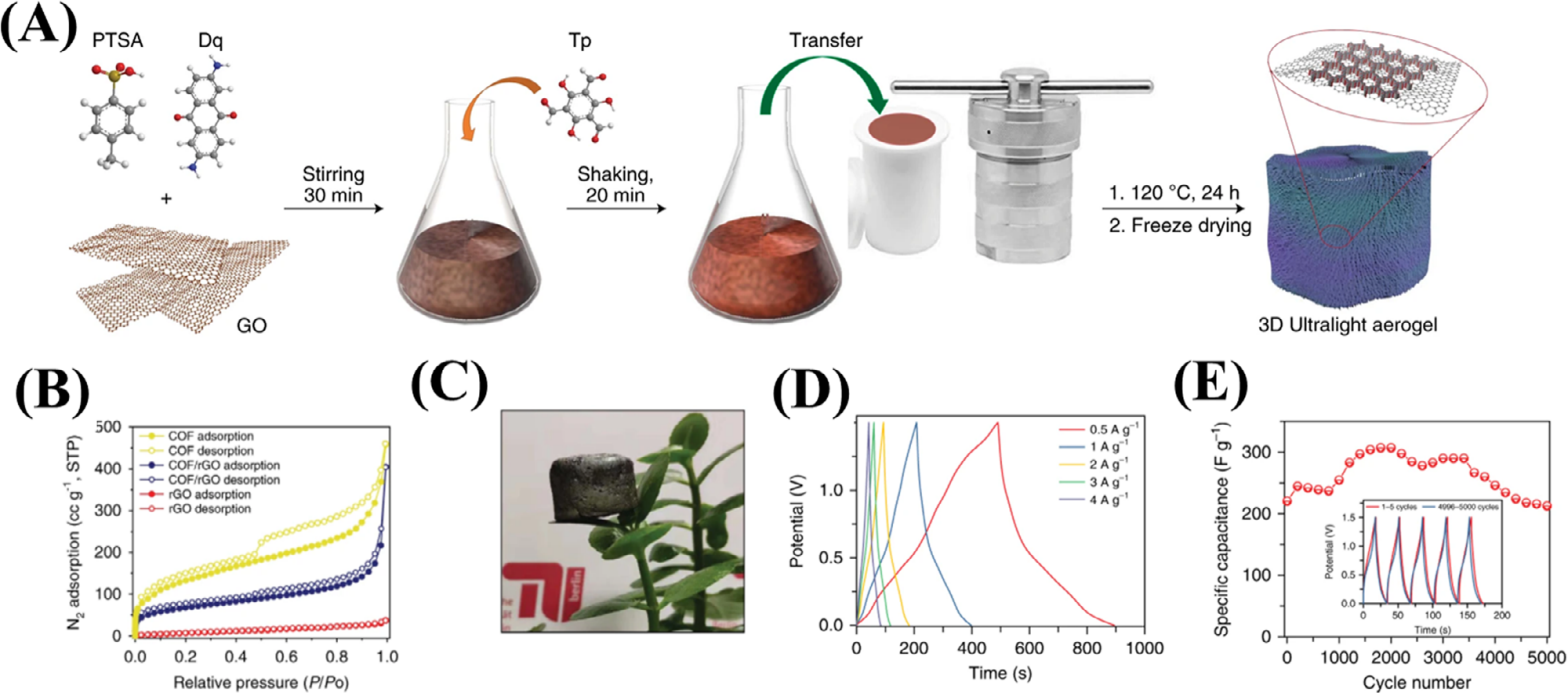
A) The synthesis procedure for covalent organic framework (COF)/rGO. B) N_2_ adsorption‐desorption isotherms of rGO, COF/rGO, and COF. C) A photograph of an ultralight COF/rGO aerogel standing on a leaf. D) The galvanostatic charge‐discharge curves of COF/rGO at a current density of 0.5, 1, 2, 3, and 4 A g^−1^. E) The cyclic stability of COF/rGO at a current density of 8 A g^−1^. Adapted with permission.^[^ ^125^ ^]^ Copyright 2020, Nature Publishing Group.

2D COFs contain a large number of mesopores, which prevent the accumulation of rGO and thus enable efficient electrolyte ion mass transfer.^[^ ^124^ ^]^ The three‐electrode configuration with the COF/rGO hybrid showed a high volumetric specific capacitance (237 F cm^−3^) and gravimetric specific capacitance (321 F g^−1^) in an aqueous electrolyte, which represented a breakthrough in capacitive graphene electrodes. In addition, efforts in constructing ultralight weight COF/rGO aerogels with stratified porosity were produced by hydrothermal treatment in the presence of GO.^[^ ^125^ ^]^ Notably, in this process, not only did the COF form a thin layer on the graphene flakes, but also the GO was reduced into rGO, and then stacked in a 3D manner to produce a COF/rGO gel (Figure 7). By taking advantage of the morphology of rGO aerogels, the COF/rGO aerogels showed a low density of about 7.0 mg cm^−3^, which was light enough to be wrapped in a leaf. The COF/rGO composites exhibited good mechanical strength and excellent electron transfer capability, and could be directly used as electrodes for SCs without the need to add conductive binders or additives. Compared with rGO and the original COFs, COF/rGO displayed a higher capacitance (269 F g^−1^ at 0.5 A g^−1^) and stronger cycle stability than most reported COFs based electrodes.^[^ ^140^, ^141^ ^]^

**FIGURE 8:**
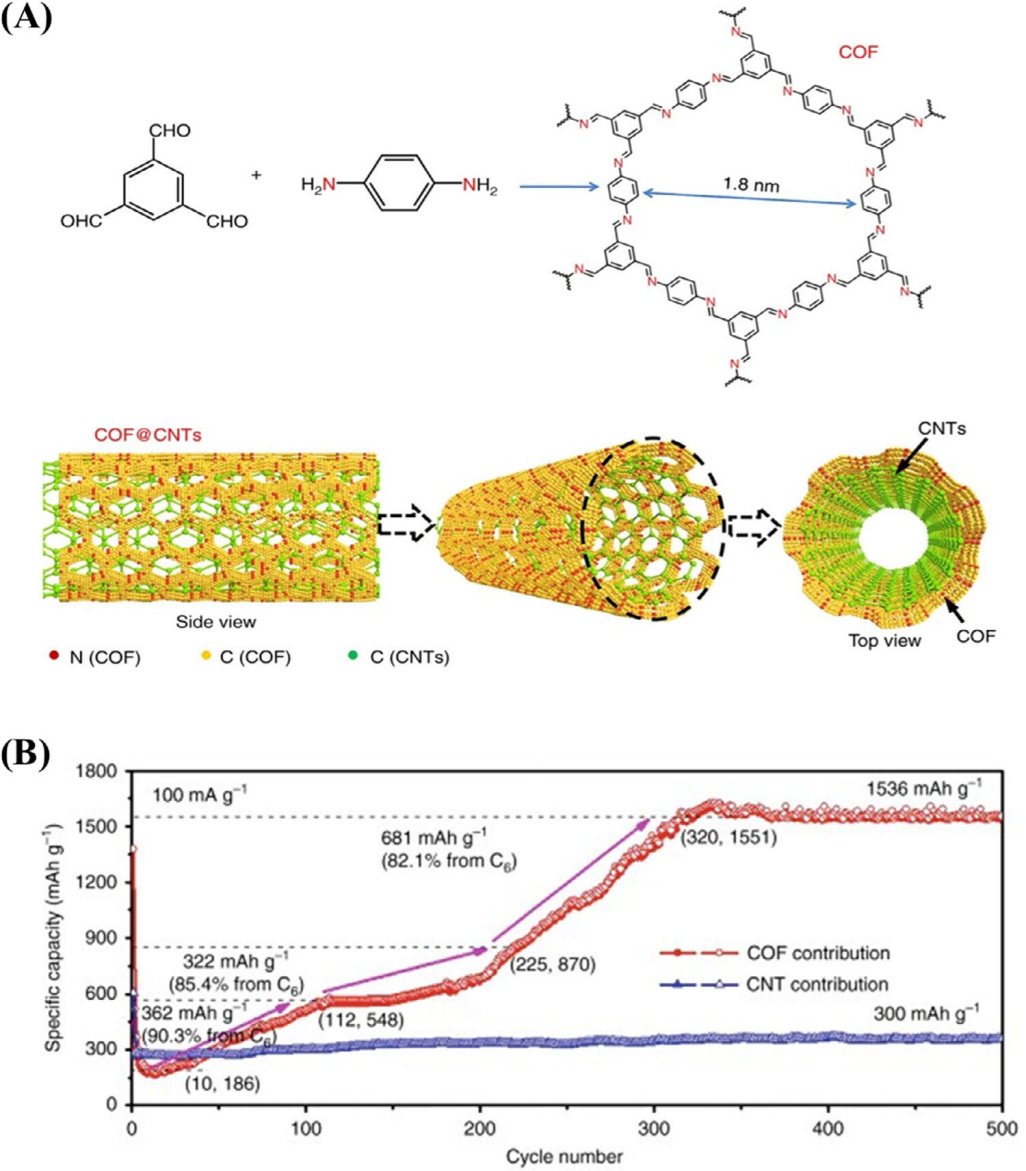
A) Preparation and B) contribution of COF@CNTs in capacity. Adapted with permission.^[^ ^29^ ^]^ Copyright 2018, Nature Publishing Group. COF, covalent organic framework.

The strong π–π interaction between COFs nano‐layer and conductive CNTs backbone can well stabilize the composites nanostructure and facilitate electron transfer.^[^ ^142^ ^]^ Similarly, wang et al. realized the growth of COFs on the CNTs surface by room‐temperature synthesis method.^[^ ^29^ ^]^ The prepared COFs@CNTs could provide a reversible capacity of up to 1536 mA h g^−1^ and maintain 500 cycles (Figure 8A). Before this, there were almost no reports of reversible capacity of 100–800 mA h g^−1^ of 2D layer COF material when LIBs were used as electrode material (Figure 8B).

In addition, rechargeable batteries such as Li‐ion batteries (LIBs), Li‐CO_2_ batteries, Na‐ion batteries, and Li‐S batteries are widely applied.^[^ ^143^, ^144^ ^]^ Due to the multiple overlapping stacking modes of COFs' strong π–π interaction, it is difficult for lithium ions to be buried in deep active sites at high current densities, making the utilization of redox active sites insufficient, reducing the lithium storage capacity.^[^ ^145^ ^]^ Mechanical exfoliation of layered organic structures (similar to graphene material exfoliated from graphite) has been considered an effective strategy to overcome this problem. Huang et al. synthesized polyimide‐COF with rGO cathode materials for lithium‐ion batteries.^[^ ^143^ ^]^ First, a mechanical grinding method was used to reduce the stacked layer numbers of COFs and shorten the ion/electron migration length. Second, rGO was chemically reduced between layers with effective redox active sites to increase charge transfer as the cathode of the lithium cell. A series of experiments indicated that incorporating rGO as well as reducing the layer number of COFs may enhance cycling stability and rate performance of COF‐based electrodes for LIBs.

In addition, using the synergistic effect of COF and CNT to grow a thin COF layer on the outer surface of CNT, the redox active sites in the DAPQ‐COF50 composites can achieve 95% utilization.^[^ ^144^ ^]^ The optimized DAPQ‐COF50 composites retain 76% capacity at 2000 mA g^−1^ after 3000 cycles, show ultra‐high rate performance, and maintain 58% capacity at 50,000 mA g^−1^. The rate capability of this material is an order of magnitude higher than all previous studies on organic electrodes containing carbonyl groups.

In 2019, Loh and colleagues became the first to construct COFs‐Ru@CNT Li‐CO_2_ cell.^[^ ^138^ ^]^ They selected COF with high stability and good CO_2_ separation performance, which was composed of the molecular units containing hydrazone/hydrazide. In addition, they combined COFs with CNTs modified by Ru NPs to construct COFs‐based cathodic catalysts (Ru‐CNTs) and found that COFs played important roles in three respects: (1) COFs act as CO_2_ traps to increase the discharge capacity; (2) COFs promote the decomposition of Li_2_CO_3_; (3) As the diffusion layer of lithium ions and CO_2_ to improve the rate performance. According to the above‐mentioned advantages, the prepared COFs‐based cathode materials show unique electrochemical property.

#### 2.3.2 Application of COFs‐carbon composites in biomedicine

As a carbon material with ultra‐small size, CDs have garnered tremendous attention from researchers in the biomedical field.^[^ ^146^ ^]^ Xie et al. combined CDs with COF, and modified them with PEG to prepare two CDs‐based COF composites, denoted as CCPF‐1@PEG as well as CCOF‐2@PEG, respectively. The prepared two kinds of nanoscale composites had good dispersibility, excellent physiological stability, and biocompatibility. And the CCOF‐2@PEG could significantly improve the generation capacity of ROS and cancer cell uptake. Both in vitro and in vivo experiments demonstrated its powerful PDT therapeutic efficiency.^[^ ^147^ ^]^

### 2.4 COFs‐sulfide composites

Differentiated structures and functions of sulfur (such as elemental sulfur, polymeric sulfur, nanosulfur, and metallic sulfides) could lead to different properties.^[^ ^148^ ^]^ COFs‐sulfide composites have been widely explored for batteries and catalysis applications.^[^ ^149^, ^150^, ^151^, ^152^, ^153^ ^]^

#### 2.4.1 Application of COFs‐sulfide composites in batteries

The effective immobilization of sulfur species is the key to overcome rapid capacity decay, low efficiency, and maintain stable recyclability, which can improve the performance of sulfur complexes in the field of catalysis and battery.^[^ ^154^, ^155^ ^]^ Wang et al. prepared pyrene‐based COF (Py‐COF)/S composites by impregnating 70 wt% sulfur into Py‐COF through a melt diffusion strategy.^[^ ^156^ ^]^ Subsequently, the prepared Py‐COF/S was successfully applied to construct Li‐S batteries with long‐term stability as well as high‐rate capacity, that was, a capacity decay rate of 0.048% per cycle with a reversible capacity of 481.2 mA h g^−1^ at 5.0 C after 550 cycles.

The large electron cloud density generated by the interaction of the conjugated π unit of the phenyl group with Li_2_S*_x_* may be an important active site for suppressing the loss of polysulfides.^[^ ^157^ ^]^ Using the π‐conjugated unit of phenyl, COFs with extended space were constructed as receivers for high‐sulfur‐loaded lithium‐sulfur batteries, which can achieve a loading capacity of 88.4%. The great advantages of Li‐S conversion platform and good nanochannels provided by COFs enable the good performance of COF‐ETTA‐ETTCA‐S.^[^ ^150^ ^]^ Studies have shown that the strong interaction between Li‐P‐S and quaternary ammonium can effectively improve the capture efficiency of polysulfides. Taking advantage of this, the quaternary ammonium cations were immobilized in the pores of COFs, and subsequently, sulfur was further loaded into the nanopores to form the COF‐Ni‐S composites. The composites effectively suppressed the LiPSs tube effect and exhibited good cycling stability.^[^ ^158^ ^]^

#### 2.4.2 Application of COFs‐sulfide composites in catalysis

Metal sulfide NPs are considered to be an efficient catalyst for multiple applications owing to their high catalytic performance, low cost as well as fitting conduction band.^[^ ^156^ ^]^ Nevertheless, metal sulfides have their own problems. For example, in addition to its propensity to photocorrose during photocatalytic processes, the application of photocatalytic hydrogen production over bulk CdS suffers from two main pitfalls: the limited active sites on its surface as well as the fast recombination of holes and photogenerated electrons.^[^ ^156^, ^159^ ^]^

Previous studies have illustrated that the synergistic interaction between metal sulfides and COFs can significantly improve the charge transfer and separation efficiency.^[^ ^152^ ^]^ Therefore, the generation of COF‐metal sulfide may be an efficient avenue to suppress electron‐hole recombination and provide more active sites. Banerjee et al. deposited CdS NPs onto 2D COF, and formed CdS‐COF hybrid. Subsequently, the hybrid acted as prototype photocatalyst for hydrogen production.^[^ ^160^ ^]^ The experiment data demonstrated that photocatalytic activity of the hybrid was increased by tenfold when the amount of COF was 1 wt% COF. In addition, Zou et al. prepared uniformly dispersed CdS NPs modified covalent triazinyl framework (CdS NPs/CTF‐1) by a facile and controllable one‐pot synthetic strategy.^[^ ^153^ ^]^ Traditional methods for the preparation of CdS include impregnation reduction, chemical deposition, and hydrothermal synthesis. These early technologies often involved complex procedures, toxic solvents, and harsh reaction conditions.^[^ ^161^, ^162^, ^163^ ^]^ Compared with the individual CTF‐1, CdS or their physically mixed counterpart, the obtained CdS/CTF‐1 had higher photocatalytic activity under visible light. This is because smaller CdS NPs are obtained by using photodeposition, resulting in more active sites. Photoelectrochemical analyses as well as time‐resolved spectroscopy indicate that CdS/CTF‐1 has better separation efficiency and higher charge transport.

**FIGURE 9:**
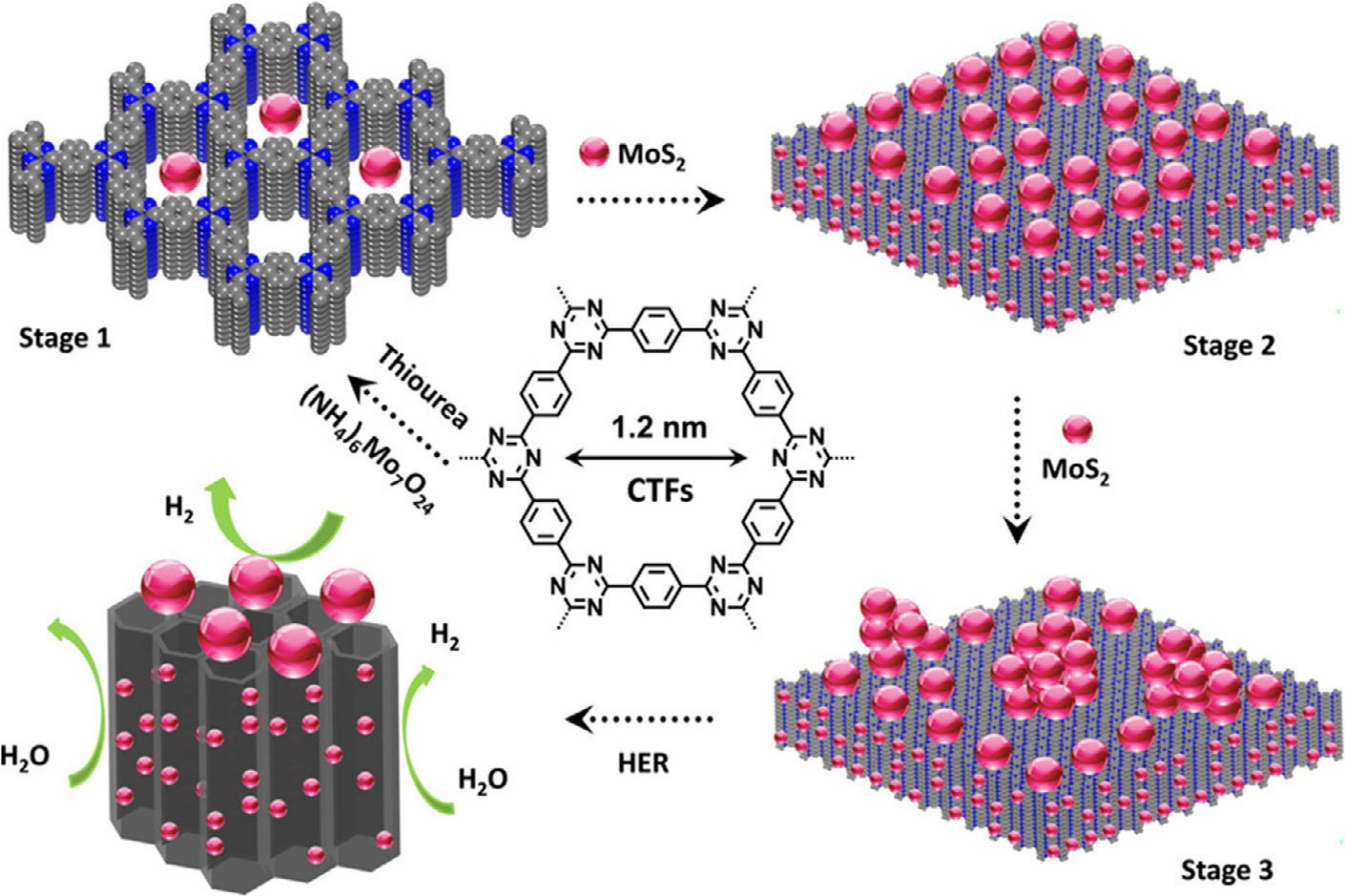
The formation process of CTFs@MoS_2_. Adapted with permission.^[^ ^164^ ^]^ Copyright 2019, Wiley‐VCH Verlag GmbH&Co.

Molybdenum disulfide (MoS_2_) nanosheets have been widely used as catalysts for the HER on account of their efficient electrocatalytic properties. Hu et al. made use of in situ growth strategy to load MoS_2_ into one‐dimensional channel of CTF to form a CTFs@MoS_2_ composites electrocatalyst, which can effectively carry out hydrogen evolution reaction on the channel or the surface of CTF.^[^ ^164^ ^]^ The π‐conjugated structure as well as the inherent confienment effect in 1 D nanochannel arrays can promote mass and electron transport. State‐of‐the‐art CTFs@MoS_2_‐5 exhibits surpassing electrocatalytic property for the HER with a small Tafel slope of 43 mV dec^−1^ and a low overpotential of 93 mV at 10 mA cm^−2^ (Figure 9).

### 2.5 COFs‐MOFs composites

Both MOFs and COFs are representative crystalline porous materials.^[^ ^165^, ^166^, ^167^ ^]^ COFs‐MOFs composites can combine the advantages of MOFs and COFs. Up to now, the synthesis strategies of composite materials based on MOFs and COFs mainly involve imine formation,^[^ ^168^ ^]^ boron‐oxygen formation,^[^ ^169^ ^]^ direct condensation,^[^ ^170^ ^]^ post‐synthetic modification,^[^ ^171^ ^]^ π–π stacking interaction,^[^ ^172^ ^]^ MOF‐in‐COF assembly strategy and modular total synthesis.^[^ ^173^, ^174^ ^]^ The combination of MOFs as well as COFs can show many superior characteristics and be widely used in many aspects.^[^ ^175^, ^176^, ^177^, ^178^, ^179^, ^180^ ^]^ For instance, Qiu and Valentin et al. proved that MOFs can be grown on the membrane of COFs to prepare COFs‐MOFs membranes.^[^ ^26^ ^]^ The as‐prepared COFs‐MOFs membranes have more excellent separation selectivity for the separation of H_2_/CO_2_ mixtures than that of COFs and MOFs alone. Table 3 summarizes the related components of typical COFs‐MOFs composites and their applications reported in recent years.^[^ ^170^, ^171^, ^172^, ^173^, ^181^, ^182^, ^183^, ^184^, ^185^, ^186^, ^187^, ^188^, ^189^, ^190^, ^191^, ^192^, ^193^, ^194^, ^195^, ^196^, ^197^ ^]^

**TABLE 3:** Summary of typical covalent organic frameworks (COFs)‐metal organic frameworks (MOFs) applications.

Precursors of COFs	MOFs	Composites	Application	Ref.
TAPB + BETH	ZPF‐2	Biomacromolecule@COF‐42‐B	Enzyme encapsulation	[181]
BTCA + TAPB	UiO‐66‐NH_2_	UiO‐66‐NH_2_@COF‐TAPB‐BTCA	Adsorption	[182]
TMT + TFPT	NH_2_‐UiO‐66	TTCOF/NUZ	Photocatalysis	[183]
Pyridine‐2,6‐dicarbonitrile + ZnCl_2_	ZIF‐67	Co_3_O_4_/N‐doped porous carbon	Electric catalytic (OER)	[184]
TFPT + DETH	NH_2_‐UiO‐66	NH_2_‐UiO‐66@TFPT‐DETH	Photocatalysis	[185]
Tp + Pa	NH_2_‐UiO‐66	NH_2_‐UiO‐66/TpPa‐1‐COF	Photocatalysis	[170]
Tp + Pa	PCN‐222‐Co	PCN‐222‐Co@TpPa‐1	Photocatalysis	[172]
TAPB + FPBA	NH_2_‐MIL‐101(Fe)	NH_2_‐MIL‐101(Fe)@NTU‐COF	Heterogeneous catalysis	[186]
1,4‐dicyanobenzene	NH_2_‐MIL‐125(Ti)	NH_2_‐MIL‐125(Ti)/B‐CTF‐1(TBC)	Photocatalysis	[171]
Tp + Pa	UiO‐66‐NH_2_	MOF@COF	Separation of CO_2_/CH_4_	[187]
Tp + Pa	ZIF‐67	MOF‐in‐COF	Separation of H_2_	[173]
COF1: Tp + Pa COF2: Tp + TPE	UiO‐66B‐NH_2_	UiO@COF1 and UiO@COF2	Identification of PO_4_^3–^and ATP	[188]
Tp + BD	Tp‐Cu‐MOF	Cu‐MOF@TPBD	Platelet‐derived growth factor‐BB (PDGF‐BB)	[189]
Terephthalonitrile	Co‐MOF	Co‐MOF@TPN‐COF	Detection of ampicillin	[190]
TP + TTA	NH_2_‐MIL‐88B(Fe)	NMC_Tp‐TTA_	Anti‐bacteria	[191]
TFPA + TAPA	NH_2_‐MIL‐68	NH_2_‐MIL‐68@COF	Detection of sulfonamides	[192]
Tp + Pa	UiO‐66‐NH_2_	UiO@TapbTp	Anti‐inflammatory drugs	[193]
Tp + Pa	NH_2_‐MIL‐125(Ti)	NH_2_‐MIL‐125(Ti)@TpPa‐1	Detection of UO_2_^2+^ and Eu^3+^	[194]
PDA + BTCA	Mn‐MOF	COF/Mn‐MOF	Lithium storage	[195]
BTCA + Pa	MOF‐UiO‐66‐NH_2_	aza‐MOF@COF	Supercapacitor	[196]
DMTP + TAPB	MOF of Bi‐Mn‐porphyrin (BM)	MOF@COF	Microwave thermal‐dynamic therapy and anti‐angiogenesis of colorectal cancer	[197]

Abbreviations: ATP, adenosine‐5′‐triphosphate; BETH, 2,5‐bis(ethoxy)terephthalohydrazide; BTCA, 1,3,5‐benzenetricarbaldehyde; DETH, 2,5‐diethoxybenzene‐1,4‐dicarbohydrazide; FPBA, 4‐formylphenylboronic acid; TAPA, tris(4‐aminophenyl)amine; TFPA, tris(4‐formylphenyl)amine; TFPT, 1,3,5‐tris‐(4‐formyl‐phenyl)triazine; TMT, 2,4,6‐trimethyl‐1,3,5‐triazine; TPA, terephthaldicarboxaldehyde; TPE, tetraamino‐tetraphenylethylene.

#### 2.5.1 Application of COFs‐MOFs composites in energy storage and conversion

COFs have been discovered to be ideal materials for various demanding energy storage and conversion applications owing to the low density, reticulated nature, as well as customizable synthesis technique.^[^ ^164^, ^198^ ^]^

**FIGURE 10:**
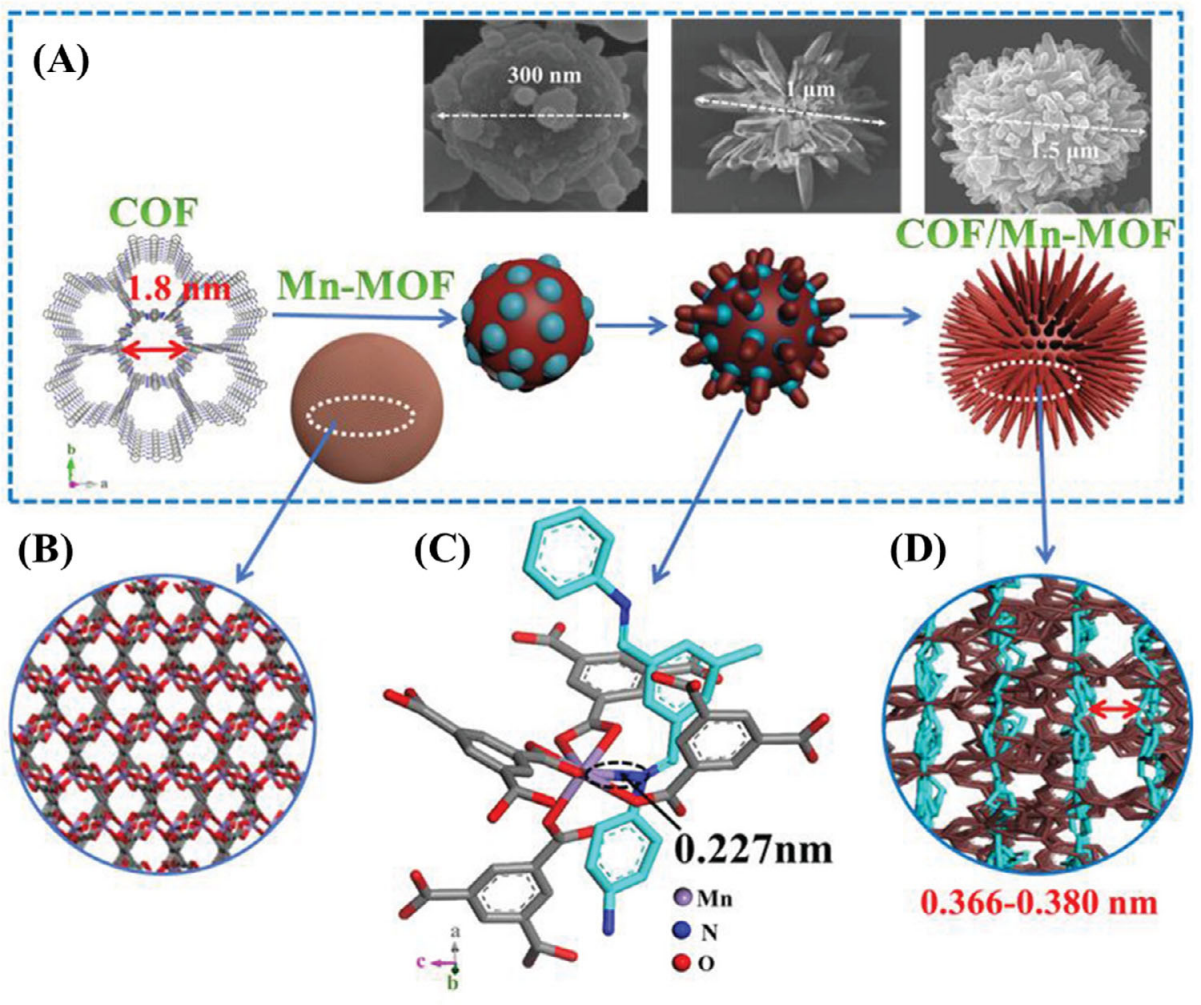
A) Schematic diagram of covalent organic framework (COF)/Mn‐metal organic framework (MOF) hybrid. B) The 3D structure of Mn‐MOF. C) The interlinked COF and Mn‐MOF units based on the Mn‐N interaction along the *c*–direction with the bonding distance of ≈0.227 nm. D) Side views of the COF/Mn‐MOF hybrid. Adapted with permission.^[^ ^195^ ^]^ Copyright 2019, Wiley‐VCH Verlag GmbH&Co.

The latest experimental breakthrough in the synthesis of 2D π‐conjugated MOF and COF composites with strong magnetic properties and excellent conductivity has drawn renewed attention to their electronic properties.^[^ ^199^ ^]^ Different from the original COFs or Mn‐MOF, Wang et al. synthesized a flower‐like COF/Mn‐MOF cross‐linked heterostructure by using coordination‐induced interlinked hybrid between metal ions in MOFs and imine groups in COFs (Figure 10).^[^ ^195^ ^]^ In this work, COFs were interlinked horizontally with MOFs, not simply bound to MOFs in the interlayer or core–shell structure. Compared with MOFs and COFs alone, the new active sites of the composites had a strong synergy. In the case of N/S co‐doped C, hollow or core–shell MnS microspheres with excellent electrochemical properties were formed. At the same time, the flower‐like COF/Mn‐MOF cross‐linked heterostructure (N/S co‐doped C) with hollow or core–shell structures had good lithium storage capacity and cycling stability. In addition, structural tuning, nano/micromorphology design, and performance optimization of porous organic framework composites at the molecular level could be elucidated by design strategies for heterogeneous structures of COFs‐MOFs composites.

**FIGURE 11:**
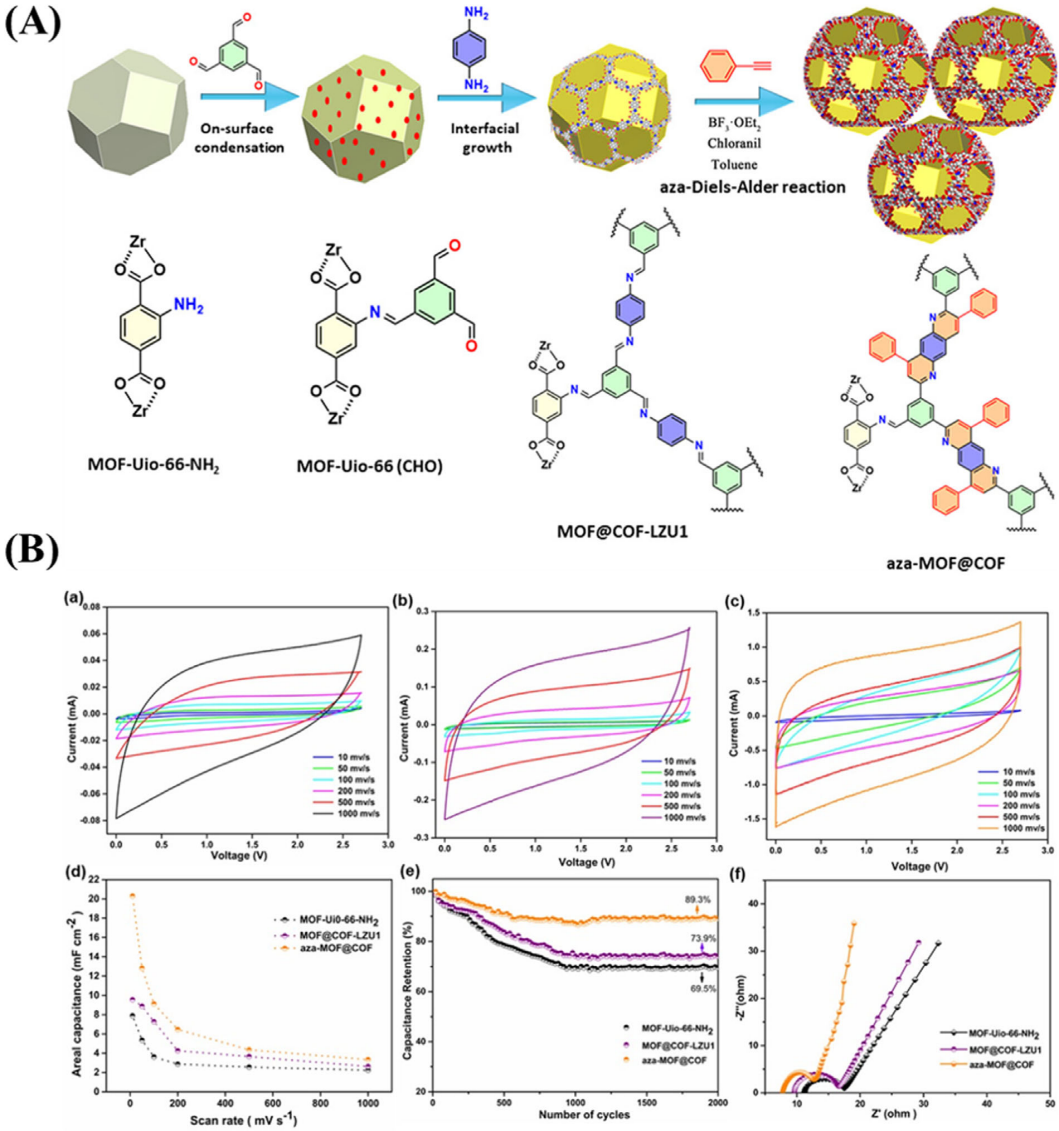
A) Schematic diagram of MOF@COF‐LZU1 synthesis. B) Cyclic voltammetry curve of a) MOF‐UiO‐66‐NH_2_, b) MOF@COF‐LZU1, c) aza‐MOF@COF, d) areal capacitance, e) capacitance retention, and f) Nyquist electrochemical impedance spectra. Adapted with permission.^[^ ^196^ ^]^ Copyright 2020, Wiley‐VCH Verlag GmbH&Co. COF, covalent organic framework. MOF, metal organic framework.

Following the utilization of Co8‐MOF‐5 by Diaz et al., MOFs were beginning to be used in supercapacitors.^[^ ^200^, ^201^ ^]^ However, the low specific capacity of MOFs hindered their application as electrodes and energy storage materials for SCs.^[^ ^196^, ^200^ ^]^ Samorì et al. recently reported an aza‐MOFs@COFs hybrid electrode material (Figure 11).^[^ ^196^ ^]^ A symmetric solid state capacitor was assembled with aza‐MOFs@COFs as electrode. Compared with pure MOF‐UiO‐66‐NH_2_ or MOF@COF‐LZU1, the cyclic voltammetry curve of aza‐MOFs@COFs based electrode showed a higher current value and more regular shape, confirming that the electrochemical stability and capacitance of this material had been improved. The results of electrochemical alternating current impedance (EIS) confirmed that the post‐synthetic modification of MOF@COF‐LZU1 improved the electrical conductivity. In addition, the slope of the second segment of the EIS curve of N is steeper than MOF@COF‐LZU1 or pure MOF‐UiO‐66‐NH_2_, indicating that ion penetration into the surrounding electrode material is increased. It can produce an area‐specific capacity of 20.35 μF cm^−2^. After 2000 cycles, the electrode still has 89.3% capacitance retention and high durability.

#### 2.5.2 Application of COFs‐MOFs composites in photocatalysis

Compared with the conventional photocatalysts TiO_2_, g‐C_3_N_4_, MOFs, COFs have better light absorption and more suitable forbidden band width. At the same time, because each atom of COFs is covalently bonded and extends to the main chain, it has excellent stability that conventional photocatalysts do not have.^[^ ^202^, ^203^, ^204^, ^205^ ^]^ However, COFs usually have low photoelectron‐hole pair mobility and fast complexation, leading to poor photocatalytic activity.^[^ ^170^ ^]^ In order to address the above‐mentioned problems and acquire more excellent performance, COFs‐MOFs composites appeared.^[^ ^206^ ^]^ The strong interaction between COFs and MOFs can effectively inhibit the recombination of electrons and holes to further improve the photocatalytic activity.^[^ ^207^ ^]^

**FIGURE 12:**
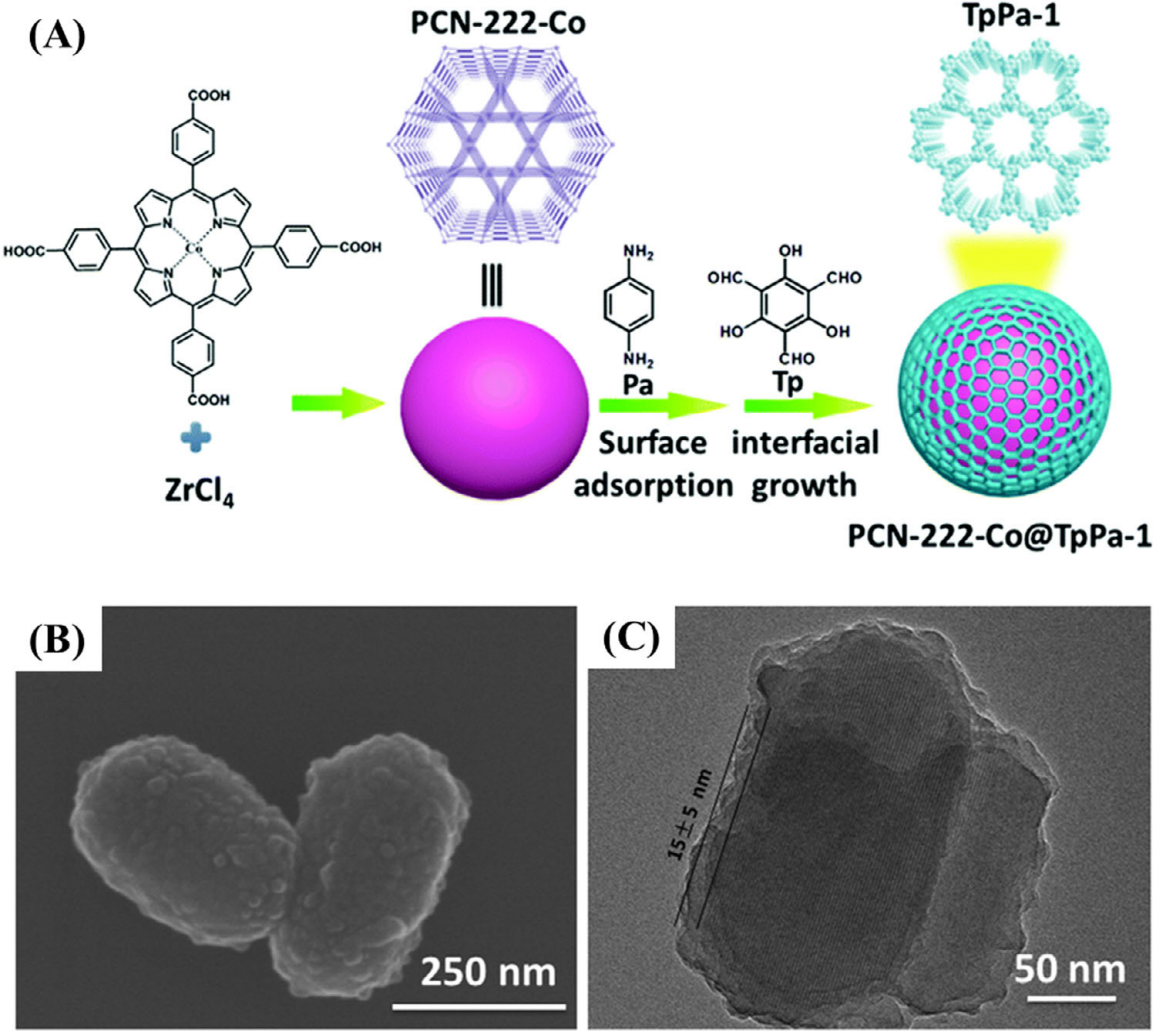
A) Synthetic process of PCN‐222‐Co@TpPa‐1. B) SEM image of PCN‐222‐Co@TpPa‐1. C) TEM image of PCN‐222‐Co@TpPa‐1. Adapted with permission.^[^ ^172^ ^]^ Copyright 2019, Royal Society of Chemistry.

NH_2_‐MIL‐68@TPA‐COF core–shell materials were first synthesized in 2017, which had excellent photocatalytic degradation performance of rhodamine B.^[^ ^35^ ^]^ This work hews out a new way for the preparation and promising applications of other COFs‐MOFs composites. Subsequently, Sun et al. successfully prepared core–shell structure MOFs@COFs (Pd/TiATA@LZU1) at room temperature without additional functionalization steps.^[^ ^204^ ^]^ Photocatalytic experiments showed a synergistic effect between the three components of the composites, which indicated that the MOF nucleus was the electron donor, the metal was the active center, and the COF shell was the electron transfer medium. Han and coworkers synthesized PCN‐222‐Co@TpPa‐1 via a strong p‐p stacking interaction (Figure 12).^[^ ^172^ ^]^ The composites can be used as an efficient catalyst for the one‐pot deacetaldehyde‐Knoevenagel condensation cascade reaction. The experimental results showed that the conversion and yield exceeded 99%, and the catalytic activity did not change significantly after 5 consecutive runs.

The emergence of “donor‐dielectric‐acceptor” systems for metal‐doped COFs‐MOFs is important for the design of photocatalysts with different efficiencies based on metal‐doped COFs‐MOFs. Ti‐MOFs@Pt@DM‐LZU1 was similarly reported, which was a sandwich‐type hydrophobic photocatalyst.^[^ ^206^ ^]^ The wettability of NH_2_‐MIL‐125 (Ti) was significantly improved by the shell of the COF. As a proof of concept, superhydrophobic Ti‐MOF@Pt@DM‐LZU1 had good photocatalytic performance for H_2_ olefins, which further indicated the combination of MOFs and COFs was an effective method to establish interfacial restricted photocatalytic layered porous composites with special wettability. Interestingly, in 2020, Wang et al. designed a novel MOF‐based In_2_S_3_‐X_2_S_3_ (X = Bi; Sb)@TFPT‐COF catalyst that had excellent photocatalytic performance under visible light.^[^ ^208^ ^]^

**FIGURE 13:**
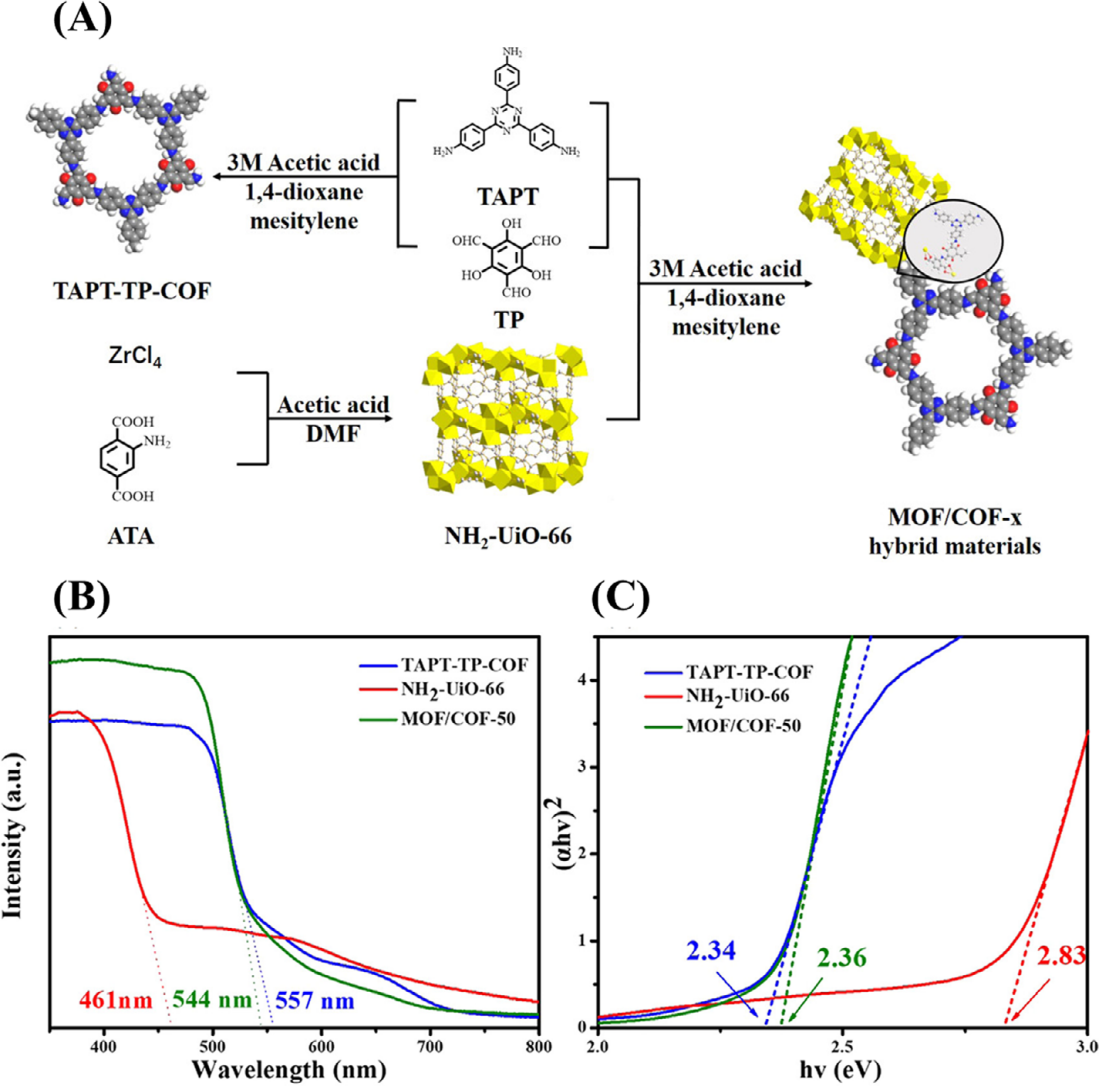
A) Schematic diagram of synthesizing metal organic framework (MOF)/covalent organic framework (COF)‐*x* hybrid materials. B) UV–vis diffuse reflectance spectra and C) the corresponding Tauc plots (the intersection of the dashed lines with the *x*–axis indicates the value of the optical band gap) of NH_2_‐UiO‐66, TAPT‐TP‐COF, and MOF/COF‐50. Reproduced with permission.^[^ ^209^ ^]^ Copyright 2021, American Chemical Society.

Another typical MOFs material is NH_2_‐UiO‐66. NH_2_‐UiO‐66@COF hybrid material has been applied to photocatalytic hydrogen production in acidic or neutral condition.^[^ ^185^ ^]^ In this work, the concept of heteroframework photocatalyst was initially proposed, and a strong covalent bond was formed between the CHO group of TFPT‐DETH and the terminal NH_2_ group of NH_2_‐UiO‐66. The prepared heteroframework photocatalyst NH_2_‐UiO‐66@TFPT‐DETH exhibited an excellent hydrogen evolution rate of 7178 μmol g^−1^ h^−1^ in water, which was comparable with or even better than those reported COFs‐based photocatalysts. But photocatalytic production of hydrogen in alkaline environments was rarely reported. As a result, the NH_2_‐UiO‐66@TAPT‐TP‐COF composites with high stability and good environmental adaptability have emerged (Figure 13A).^[^ ^209^ ^]^ Through characterization, it can be found that the composites have significantly red‐shifted than NH_2_‐UiO‐66 or TAPT‐TP‐COF alone (Figure 13B). The obvious red shift shows a strong absorption ability of visible light, and the matching band potential between NH_2_‐UiO‐66 and COF enables photogenerated charges to transfer, thereby effectively separating photogenerated electrons from holes (Figure 13C).

#### 2.5.3 Application of COFs‐MOFs composites in biomedicine

**FIGURE 14:**
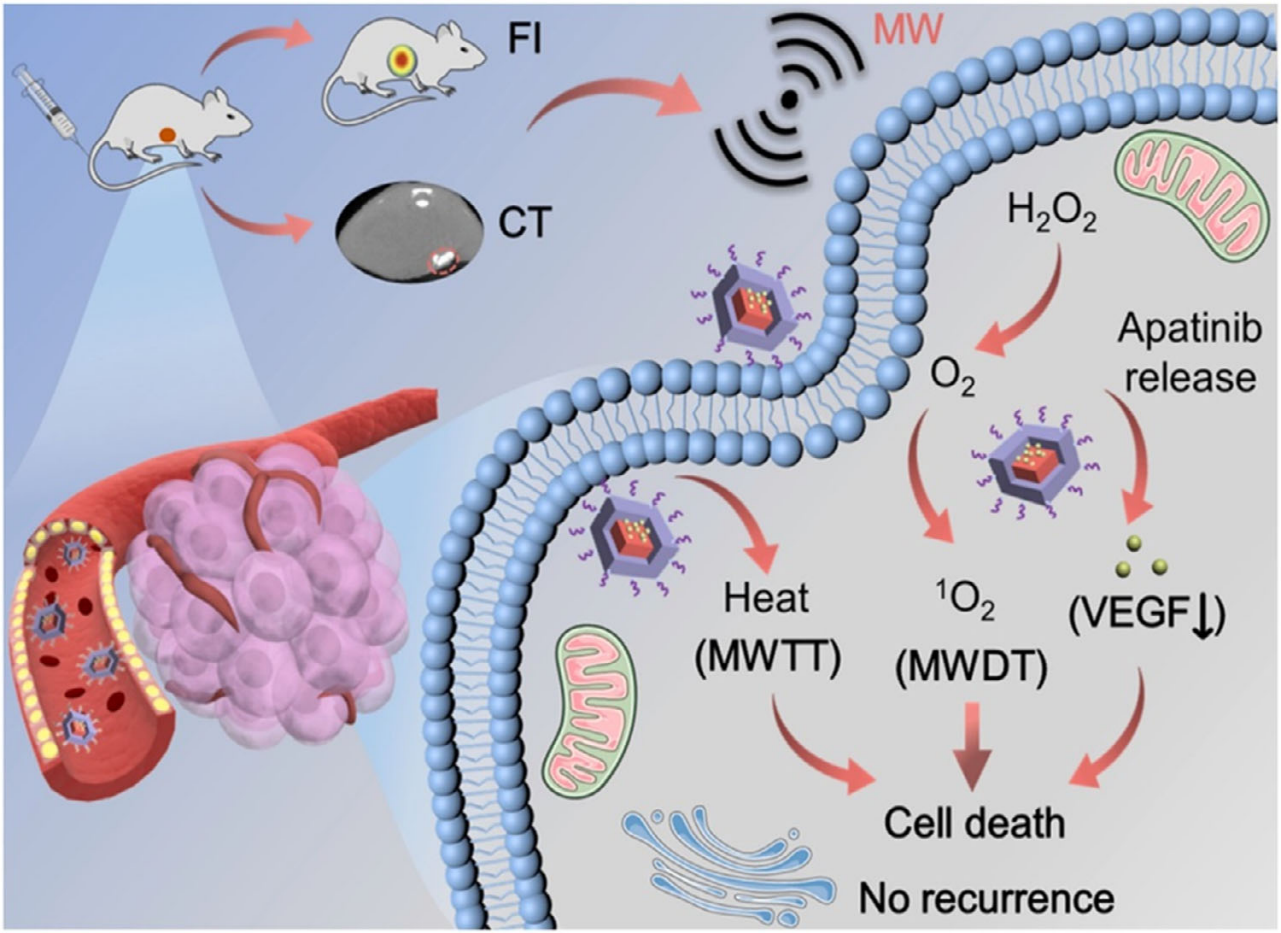
Illustration of BMCAP nanocapsules for microwave dynamic thermotherapy (MWDT‐MWTT) and anti‐angiogenesis in colorectal cancer. Reproduced with permission.^[^ ^197^ ^]^ Copyright 2019, Elsevier.

Tumor recurrence is a major disease that harms human health more than the original malignant tumor.^[^ ^210^ ^]^ In addition to traditional treatment approaches such as chemotherapy as well as drug therapy, microwave induction therapy has the advantage of deep penetration depth, not easy to be disturbed by bones and gases, and has played a huge role in anti‐tumor recovery.^[^ ^211^ ^]^ To this end, a new MOFs@COFs nanocapsule was developed for the enhanced microwave hyperthermia and dynamic therapy (Figure 14).^[^ ^197^ ^]^ This nanocapsule could generate heat for microwave hyperthermia through the ion confinement effect of nanoporous structure under microwave irradiation. Due to the separation of electron‐hole pairs in the system containing transition metal Mn, it could also be stimulated by microwaves to generate ^1^O_2_ for microwave dynamic therapy, which had an inhibitory effect on tumor recurrence after microwave therapy.

Recently, inspired by nanoenzymes,^[^ ^212^ ^]^ the first COFs‐MOFs nanoenzyme was constructed with sufficient active sites in a customized microenvironment and surface to enhance the catalytic activity and therapeutic efficiency of the enzyme.^[^ ^191^ ^]^ COF_Tp‐TTA_ is formed with a specific surface functional group and acts as a binding pocket for the amino‐functionalized peroxidase enzyme (NH_2_‐MIL‐88B(Fe)), providing the composites with improved catalytic properties over a wide range of pH. The pseudopodia‐like surface of the COF “skin” enabled it to catch the bacteria effectively and efficiently kill the bacteria by ROS generated in situ. Generally, COFs‐MOFs composites offer new insights into the treatment of various diseases.

### 2.6 COFs‐enzymes composites

Enzyme‐based biocatalysts have the advantage of excellent catalytic properties, eco‐friendly reaction mechanisms, and high selectivity.^[^ ^213^, ^214^, ^215^, ^216^, ^217^ ^]^

In order to keep the activity, enzyme immobilized in the holes of the silica is already a mature research field.^[^ ^215^, ^218^, ^219^ ^]^ Recently, COFs as new support matrices for the immobilization of enzymes, have offered new avenues to researchers because of their fascinating properties.^[^ ^213^ ^]^ Generally, COFs‐based enzyme biocomposites can be conveniently classified according to how they are composed. They can be classified as followed, (1) Surface binding: the enzyme is immobilized on the presynthesized COF surface through noncovalent or covalent interactions;^[^ ^220^ ^]^ (2) Pore infiltration: the enzyme is introduced into the pore network of the presynthesized COF;^[^ ^221^ ^]^ (3) Encapsulation: the enzyme is encapsulated in COF crystals.^[^ ^213^, ^214^ ^]^

**FIGURE 15:**
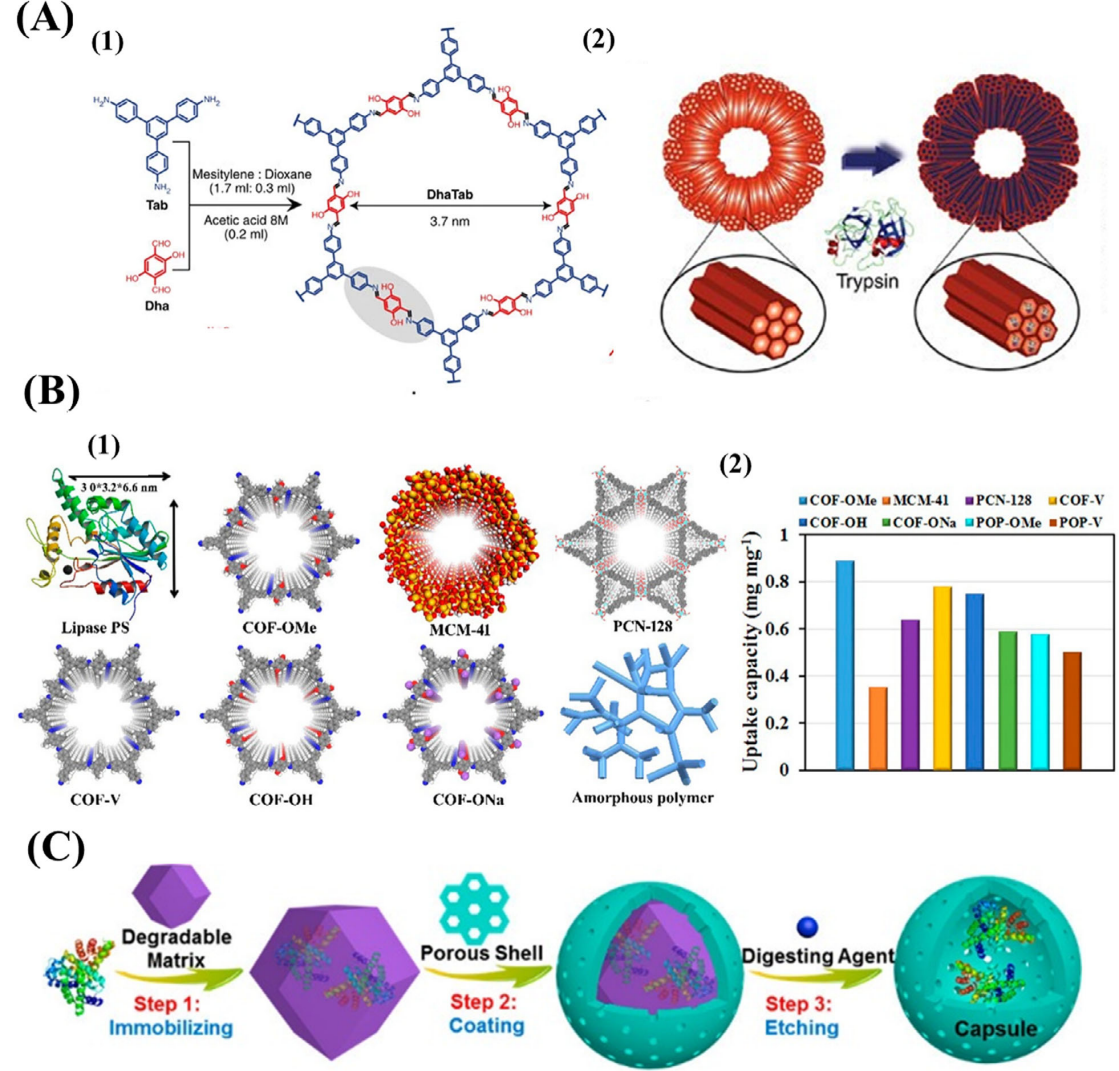
A) 1) Synthesis of covalent organic framework (COF)‐DhaTab by the Schiff base reaction of Dha and Table 2. Schematic representation of trypsin adsorption in COF‐DhaTab. Adapted with permission.^[^ ^222^ ^]^ Copyright 2015, Nature Publishing Group. B) 1) Graphic view of lipase PS and porous materials used for the immobilization of enzymes; 2) Enzyme uptake capacity of various porous materials after incubation in lipase PS solution (30 mg mL^−1^) for 6 h. Adapted with permission.^[^ ^224^ ^]^ Copyright 2018, American Chemical Society. C) Stepwise approach to construct biomacromolecule@capsule. Adapted with permission.^[^ ^181^ ^]^ Copyright 2020, American Chemical Society.

The most common method of enzyme immobilization is surface binding.^[^ ^214^ ^]^ Banerjee et al. made a pioneering attempt to construct a COFs‐enzyme biological conjugated system by adopting an adsorption methodology. The process can be completed in one step without any template.^[^ ^222^ ^]^ This material was used to immobilize trypsin, and about 60% of the free trypsin remained active, indicating the usefulness of COFs as the correct platform for enzymes. Initial studies have shown that enzymes can be effectively immobilized to COFs. However, the activity of the immobilized enzyme decreased compared with free enzyme. It is inferred that the substrate is inaccessible to the enzyme in the COFs microenvironment, which affects the enzyme activity (Figure 15A).

Although the immobilization of enzymes by physical adsorption is easily implemented, the mutual properties of the molecules may lead to the leaching of the enzymes, making them less efficient during recycling.^[^ ^223^ ^]^ Sun et al. proved that COFs could provide an excellent microenvironment for enhancing enzyme activity. Compared with other porous materials such as MOFs or mesoporous silica, COFs not only made the accommodated enzymes more accessible to the reagents but also served as stronger shields to safeguard the enzymes from deactivation. (Figure 15B).^[^ ^224^ ^]^ The established trypsin bioreactor has been successfully used for enzymatic digestion of model protein, and its digestion efficiency has been significantly improved. The results showed that the process could be completely digested in only 2 min, and had the advantages of good stability and repeatability. Recently, an excellent method of encasing enzymes in the COF capsules using digestible MOF as a template was described for constructing hollow COF capsules.^[^ ^181^ ^]^ In the clever strategy, enzymes were attached to MOFs to form biomacromolecule@MOF biological complexes, which was the prototype for growing biomacromolecule@MOF@COF core–shells and protecting biological macromolecules (Figure 15C).

Polystyrene spheres are used as templates to synthesize COFs, which can form interconnected nanopores within the COFs, and convert enzymes (glucose oxidase and horseradish peroxidase as a model) encapsulated into the nanopores of COFs.^[^ ^225^ ^]^ The nanoreactors offer faster mass transport and more accessible active sites compared to the single‐pore structure, which significantly improves the catalytic performance of internal enzyme and shows protection of enzyme in harsh environment.

### 2.7 COFs‐oncology drugs composites

Nanomaterials are enriching biomedical science to a certain extent, including providing suitable drug delivery systems for tumor therapy, bioimaging, and biosensing agents.^[^ ^226^ ^]^

In the past decade, the development of drug delivery has promoted the successful application of many drugs in the treatment of diseases.^[^ ^227^ ^]^ Drug delivery technologies can enhance targeted drug delivery, minimize drug off‐target effects, and improve patient compliance.^[^ ^216^, ^228^ ^]^ As the therapeutic research of oncology drugs evolves from small molecules to nucleic acids, proteins, peptides, and antibodies, drug delivery technologies continue to evolve to meet the new challenges that come with it.^[^ ^76^ ^]^ At the same time, COFs have been proved to be metal ion‐free porous materials with less biotoxicity than MOFs, with large specific surface area and high porosity, and can be acted as carriers for drug delivery. The research related to COFs is on the rise.^[^ ^219^, ^228^ ^]^

**FIGURE 16:**
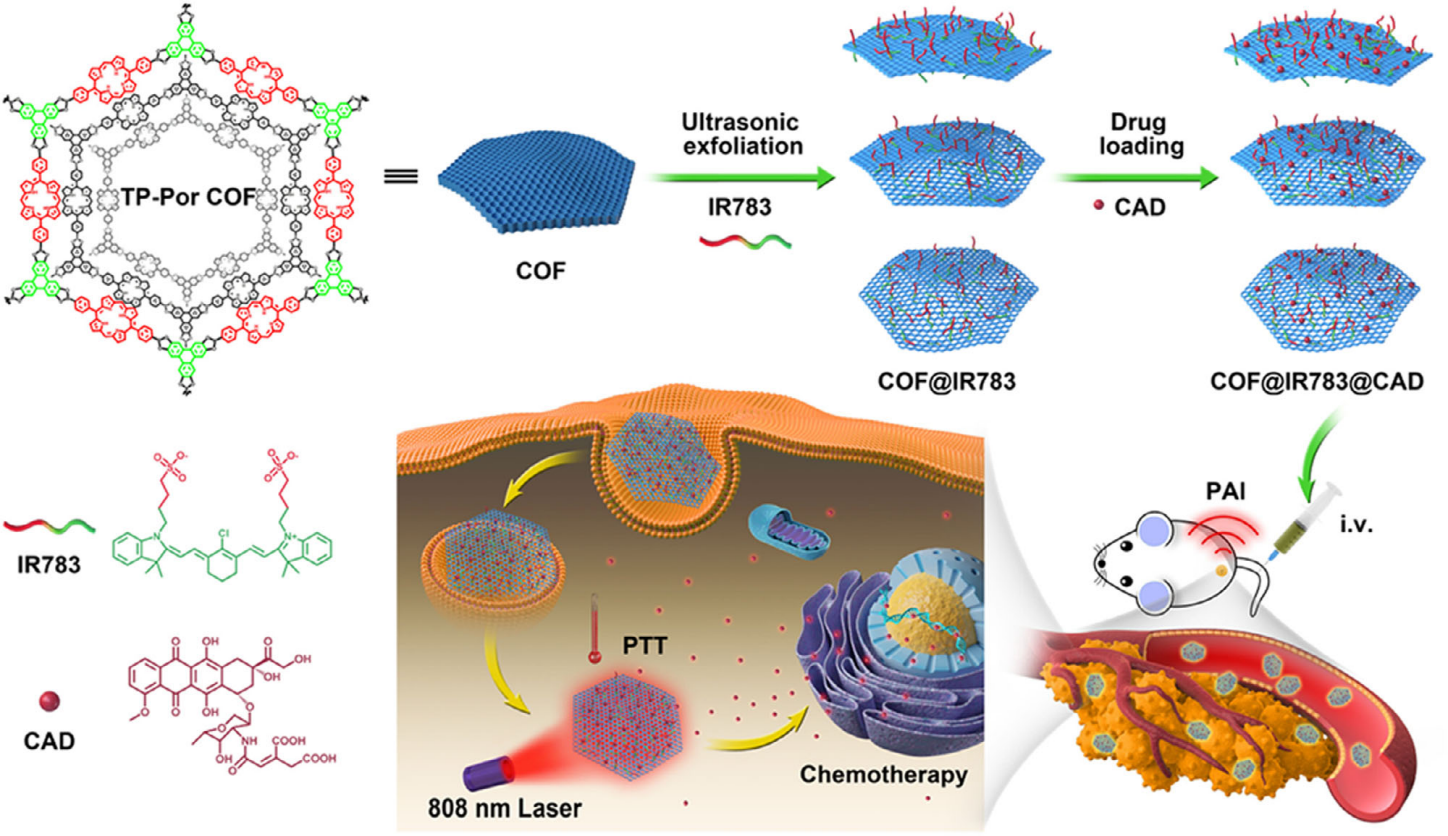
Schematic illustration of the nanocomposites fabrication, drug delivery, and in vivo combinative antitumor therapy. Adapted with permission.^[^ ^229^ ^]^ Copyright 2019, American Chemical Society.

Chen et al. synthesized a water‐dispersible multifunctional COF@IR783 composite by using cyanine as a stabilizer.^[^ ^229^ ^]^ On the one hand, the formed COF@IR783 dispersion was suitable for in vivo. Meanwhile, the dispersion also possessed improved PTT capability in the near‐infrared region and photoacoustic imaging ability. With its unique biodegradability and π–π interactions, it can be used to bind precancerous drugs, making COFs composites shine in drug delivery (Figure 16).

Hypoxic tumor microenvironment is a significant impediment to good PDT efficacy. Zhang et al. prepared functional COFs that could promote PDT performance via remodeling tumor extracellular matrix.^[^ ^230^ ^]^ After intravenous injection, the composites can accumulate and release anti‐fibrotic drug at the tumor site and effectively improve the tumor's oxygen‐depleted microenvironment. In addition, PCPP‐mediated tumor extracellular matrix remodeling also enhanced tumor cell uptake of subsequently injected protoporphyrin IX (PPIX)‐conjugated peptide NPs (NM‐PPIX), and synergistically enhanced the response to PDT. Ca^2+^ are ubiquitous yet subtle regulators of cellular physiology. However, intracellular Ca^2+^ uptake and export are a highly controlled and naturally occurring process.^[^ ^231^ ^]^ Due to the poor stability of CaO_2_ in the physiological environment, Ca^2+^ signaling can even promote the survival of cancer cells.^[^ ^232^ ^]^ Inspired by this work, Dong et al. designed and synthesized an NCOF‐based nanoagent of a CaCO_3_@COF‐BODIPY‐2I@GAG (4) composites using the principles of Ca^2+^ overload and PDT process, where BODIPY‐2I as a photosensitizer (PS) CaCO_3_ NPs can be safely delivered to the region of tumor cells (HCT‐116) via endocytosis by targeting of the targeting agent (GAG) under the protection of COF.^[^ ^233^ ^]^ PDT can produce irreversible damage to cells, losing the ability to control Ca^2+^ when the release of Ca^2+^ can be achieved. Guinness is able to kill tumor cells more effectively because of their synergistic effect of PDT as well as Ca^2+^ overload.

Researchon COF‐based composites of tumors is still at a preliminary stage, probably due to (1) the poor dispersion and aqueous stability of COFs itself, and the drug loading is not easy to be absorbed by cell;^[^ ^229^ ^]^ (2) The biosafety of COF needs to be further evaluated; (3) The synthesis cost of COF is high, and it is difficult to be mass produced.^[^ ^216^ ^]^

### 2.8 Other COFs‐based composites

Other materials, such as metal cluster, polymer, SiO_2_, MXene, g‐C_3_N_4_, ionic liquids (ILs), and quantum dots (QDs) have also been composited with COFs for various applications.^[^ ^31^, ^203^, ^234^, ^235^, ^236^ ^]^ Here we make statistics on other types of COFs‐based composites in Table 4.^[^ ^31^, ^48^, ^234^, ^235^, ^236^, ^237^, ^238^, ^239^, ^240^, ^241^, ^242^, ^243^, ^244^, ^245^, ^246^, ^247^, ^248^, ^249^, ^250^, ^251^, ^252^, ^253^, ^254^, ^255^, ^256^, ^257^, ^258^ ^]^

**TABLE 4:** Summary of typical examples of other covalent organic frameworks (COFs)‐based composites.

Precursors of COFs	Functional materials	Composites	Application	Ref.
Tp + Pa	Polymer substrate	TpPa/Polysulfone	Separation of molecular	[31]
Tp + Pa	CdSe/ZnS QDs	QDs‐grafted COFs	Protein sensing	[48]
Pa + HTA Pa + DHTA Pa + THTA	NH_2_‐Ti_3_C_2_T*_x_*	COF/NH_2_‐Ti_3_C_2_T*_x_*	Photocatalysis	[234]
Tp + BD	g‐C_3_N_4_	COF/g‐C_3_N_4_	Photocatalysis	[235]
TAPB + Dva	Au cluster	Au@COF	photocatalysis	[236]
2,2′‐bipyridyl + 1,5‐cyclooctadiene	CNi_2_P	COP‐TF@CNi_2_P	Photocatalysis	[237]
TAPB + BTCA	LiCl	LiCl@RT‐COF‐1	Fuel cell	[238]
BTCA + Pa	Ti_3_C_2_T*_x_* nanosheets	NH_2_‐Ti_3_C_2_T*_x_* @COF‐LZU1	Li‐S batteries	[239]
DCB	Ti_3_C_2_ nanosheets	S@CTF/TNS	Li‐S batteries	[240]
Tp + Pa	Ti_3_C_2_T*_x_* nanosheets	Dual‐layered COF/MXene	Water treatment	[241]
BTCA + Pa	Ti_3_C_2_T*_x_* nanosheets	Ti_3_C_2_T*_x_*/COF‐LZU1	Separation of dye	[242]
Tetra‐(4‐anilyl)‐methane + terephthaldehyde	[bmim][Tf_2_N] ILs	IL@COF‐300	Separation of CO_2_/CH_4_	[243]
BPDA + TAM	[Emim][Tf_2_N] ILs	[Emim][Tf_2_N] IL@COF‐320	Catalysis reaction and gas separation	[244]
H_2_P + DHTA	1‐alkyl‐3‐methylimidazolium‐based ILs	AMIMBr@H_2_P‐DHPh COF	Catalysis cycloaddition reaction	[245]
TAPB + DVP + DMTP	ILs	PIL‐COF	Catalysis	[246]
TDA + TP	Imidazolium‐based ionic polymer	ImIP@TT‐COF	Catalytic CO_2_ cycloaddition	[247]
Tp + TtA	g‐C_3_N_4_/CNT	COF/g‐C_3_N_4_/CNT	Photocatalysis	[248]
Tta + Tp	g‐C_3_N_4_	g‐C_3_N_4_(NH)/COF	Photocatalysis	[249]
Tp + TTA	SiO_2_	Pd single atoms/clusters TP‐TTA/SiO_2_	Photocatalysis	[250]
TFPA + BTCA	ILs	petal‐shaped ILs‐COFs	Determination of general anesthetics	[251]
Dha + TAPB	[APMIM]Br ILs	DhaTAPB‐COF‐ EuIL	Detection of acetone	[252]
Dha + TAPB	PEG	PEG@COF	Separation of CO_2_	[253]
CC + TPB	SiO_2_	SiO_2_@CTP COF	Chromatographic separation	[254]
Tp + BD	SiO_2_	CTpBD@SiO_2_	Enantioseparation	[255]
Am7CD + PDA	SiO_2_	β‐CD‐COF@SiO_2_	Enantioseparation	[256]
DHBD + PY‐CHO	Pt cluster	Pt loaded PY‐DHBD‐COF	Photocatalysis	[257]
TFPT + HZ	PdIn bimetallic nanoclusters	PdIn@N_3_‐COF	Photocatalysis	[258]

Abbreviations: [APMIM]Br, 1‐(3‐aminopropyl)–3‐methylimidazolium bromide; BPDA, 4,4´‐biphenyldicarboxaldehyde; BTCA, 1,3,5‐benzenetricarboxaldehyde; CC, cyanuric chloride; DCB, 1,4‐dicyanobenzene; Dha, 2,5‐dihydroxyterephthalaldehyde; DHTA, 2,4‐dihydroxybenzene‐1,3,5‐tricarbaldehyde; DHPh, 2,5‐dihydroxyterephthalaldehyde; H_2_P, 5,10,15,20‐tetrakis(p‐tetraphenylamino)porphyrin; HTA, 2‐hydroxybenzene‐1,3,5‐tricarbaldehyde; HZ, hydrazine hydrate; ILs, ionic liquids; PY‐CHO, 1,3,6,8‐tetra(4‐formylphenyl)pyrene; TAM, tetra‐(4‐anilyl)‐methane; TDA, 1H‐1,2,4‐triazole‐3,5‐diamine; THTA, 4,6‐trihydroxybenzene‐1,3,5‐tricarbaldehyde; TPB, 1,3,5‐triphenylbenzene; TTA, 4,4′,4″‐(1,3,5‐triazine‐2,4,6‐triyl)trianiline.

As a promising metal cluster support, COF has also been a concern in recent years. In 2020, the raw materials of Au nanoclusters (Au NCs) were introduced into the pores of COFs, and ultra‐small and uniform Au NCs (1.8 ± 0.2 nm) were grown in situ.^[^ ^236^ ^]^ The poor stability of in situ illumination Au NCs for a long time was solved by using sulfur chains as bridges for electronic transmission. The powerful S‐Au binding is favorable to stabilize the Au NCs. Z‐scheme photocatalytic system was constructed by forming Au─S─COF bonds, which had the benefits of improving the charge separation efficiency. Through this ingenious design, a novel Au NCs/COFs composites photocatalytic system was built successfully. The mechanism of the photoelectrochemical process between Au NCs and the COFs supports was evaluated deeply, and these results showed that the degradation rate of rhodamine B by Au@COF was significantly higher than that by COFs alone, which was increased from 5.79 × 10^−2^ to 9.04 × 10^−2^ min^−1^ after 30 min of visible light irradiation. After 5 cycles, the removal rate remained above 95.4%. The obtained Au@COF was stable and showed high photocatalytic activity.

Recently, composites based on COFs have gradually expanded to membranes for efficient and precise separation.^[^ ^259^, ^260^, ^261^ ^]^ In 2020, ploymer integrated COFs packing for gas separation was reported.^[^ ^253^ ^]^ Hybrid matrix membranes were fabricated by filling COFs hollow microspheres with PEG modification into commercial pebax polymers. The hollow structure of the COF filler reduced the mass transfer resistance. The selectivity of dissolution and diffusion was achieved by PEG functionalization to reduce the pore size of COF and the hollow structure of the filler, thus reducing the transport resistance. The resulting matrix membranes exhibited excellent CO_2_/CH_4_ separation performance, exceeding the upper limit of Robeson's in 2008. In order to acquire a stable membrane with long‐term separation performance, polybenzimidazole, polythiosemicarbazide, polyimide, polyethersulfone, and other polyazoles have been used to form COFs‐based composites.^[^ ^262^ ^]^

**FIGURE 17:**
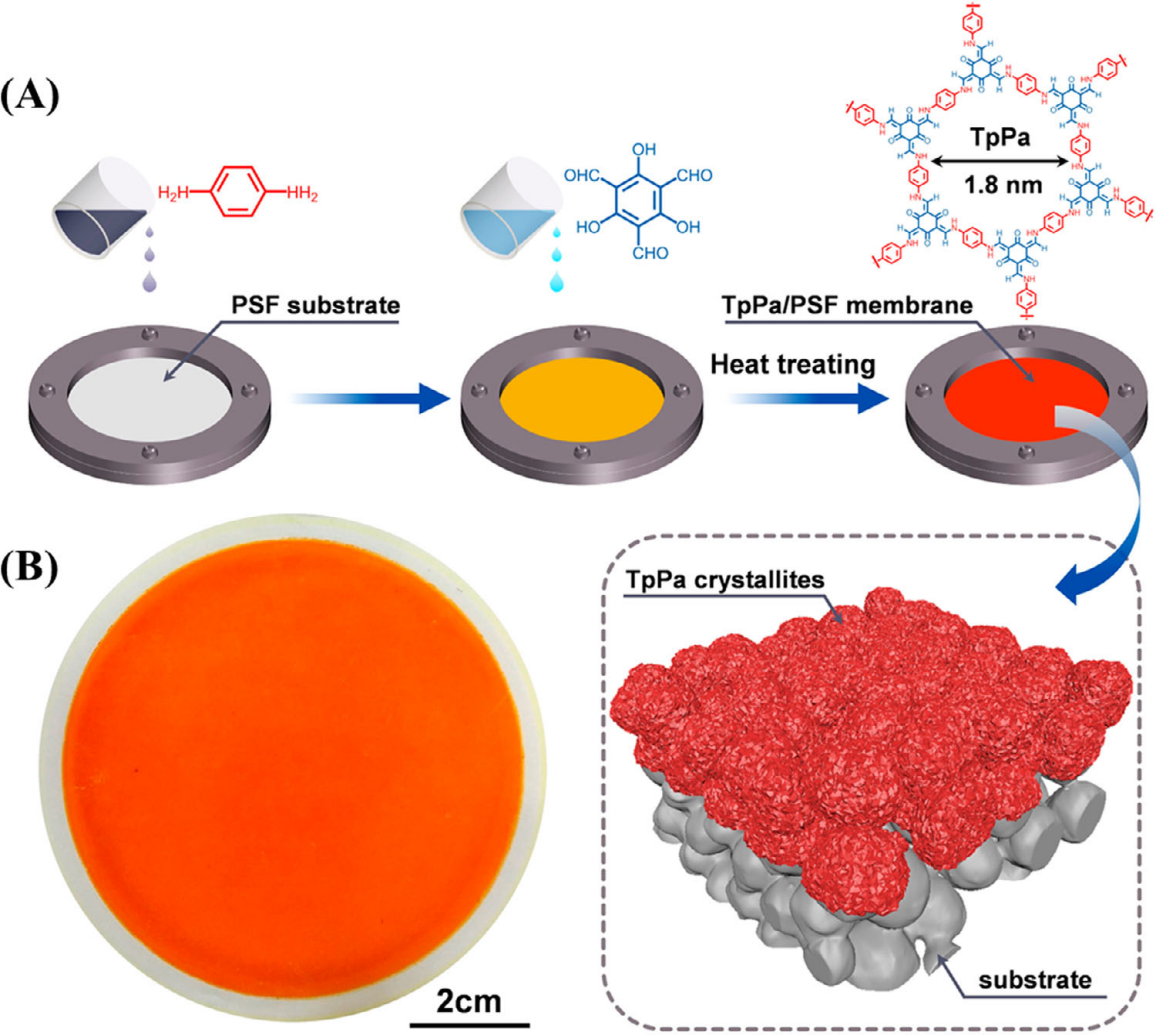
The process for preparing TpPa/PSF membranes via IP. A) Schematic representation of the TpPa/PSF composites membrane. B) Physical appearance of the obtained TpPa/PSF composites membrane. Adapted with permission.^[^ ^31^ ^]^ Copyright 2019, Elsevier.

A 2D COF (TpHZ‐8) onto a polyethersulfone (PES) substrate was constructed by a nonsolvent‐induced phase separation method for gas separation.^[^ ^218^ ^]^ The membrane separation factor reaches 1430 when used for water/ethanol separation, which is 6‐fold higher than that of pure PES membranes. The increased performance could be attributed to the functional gradient of the membranes, and the formation of water channels owing to the hydrogen bonding interaction between H_2_O and TpHZ‐8. The membrane exhibits long‐term operational stability of up to 320 h due to its good thermal and chemical stability. Wang et al. proposed a simple and easy strategy, namely interfacial polymerization, which directly synthesized imine‐linked COFs on the polysulfone substrates to produce composites membranes (Figure 17).^[^ ^31^ ^]^ Due to the moderate reaction rate between monomer pairs in corresponding organic and aqueous phases, COFs were formed rapidly within 1 min, and grew conformally along the upper surface of the substrate pores. The number of COFs had proved to be a key factor in the production of highly permeable composites membranes. Under the optimal experimental conditions, the composites membranes achieved significant H_2_O permeability up to 50 L m^−2^ h^−1^ bar^−1^ as well as high rejection (> 95%) of different dye molecules.

The particle size distribution, morphology, and homogeneity of the COFs have a considerable impact on the separation performance of HPLC column.^[^ ^263^ ^]^ From the aspect of studies, a few attempts have been made to select COF as a new kind of stationary phase for HPLC separations.^[^ ^88^, ^264^, ^265^, ^266^, ^267^, ^268^, ^269^ ^]^ By fixing different COFs with irregular shape, inhomogeneity, and low density on the surface of SiO_2_ to form composite materials, good separation can be achieved at low column back pressure.^[^ ^264^ ^]^ Fan et al. constructed an in situ growth strategy, in which COF5 was modified onto SiO_2_ NPs as a stationary phase for HPLC to form a COF‐silica composites (SiO_2_@COF5), which overcame the disadvantages of irregular COF morphology and the low column efficiency of submicron‐level COF and promoted its application in the field of HPLC.^[^ ^267^ ^]^ In order to increase the separation performance, Zhang et al. fabricated core–shell SiO_2_@covalent triazine‐based organic polymer (CTP) microspheres for mixed‐mode chromatographic stationary phase.^[^ ^254^ ^]^ Multiple retention mechanisms (such as π–π, electron donor‐acceptor, hydrophobic, hydrogen‐bonding interactions) could be provided by this stationary phase due to the synergistic interaction of triazine and aromatic groups on CTP. Based on the above‐mentioned interaction mechanisms, the successful separation of several hydrophilic and hydrophobic analytes with high shape selectivity is achieved by performing different chromatographic conditions. COF‐LZU1 (Lan Zhou University‐1) is a “star” of imine bonded COFs. COF‐LZU1 is stable in water and most organic solvents, rich in multiple benzene rings, and providing a rich conjugated structure. Therefore, the SiO_2_@rLZU1 (reduced Lan Zhou University‐1) composites acted as a new reversed‐phase chromatographic stationary phase, which exhibited good reproducibility and high resolution in the separations of acidic, neutral, and basic compounds.^[^ ^268^ ^]^

Chiral COFs are a fascinating separation medium in HPLC.^[^ ^264^ ^]^ In 2021, monodisperse chiral CTpBD@SiO_2_ microspheres were fabricated as a chiral stationary phase for enantioseparation via an in situ growth strategy.^[^ ^255^ ^]^ The chiral COF@SiO_2_ microspheres integrate the excellent column packing properties of silica and the unique chiral recognition ability of chiral COF. The results indicated that the CTpBD@SiO_2_‐coated columns had excellent chiral separation performance for ketones, phenols, alcohols, bases, amines, and other chiral compounds. In addition, a facile one‐pot method is reported to prepare a novel β‐CD‐COF@SiO_2_ chiral stationary phase for enantioseparation.^[^ ^256^ ^]^ The prepared β‐CD‐COF@SiO_2_ stationary phase exhibited high enantioselectivity and excellent separation performance for diverse racemic compounds.

In another report, composites were prepared successfully by growing chiral COFs (BtaMth COFs) on the surface of SiO_2_‐NH_2_ spheres.^[^ ^264^ ^]^ The synthesized BtaMth@SiO_2_ composites exhibited excellent resolution for the separation of the *cis*‐*trans* isomers of β‐cypermethrin and metronidazole in normal‐phase mode and the positional isomers of nitrochlorobenzene and nitrotoluene in reversed‐phase mode.

**FIGURE 18:**
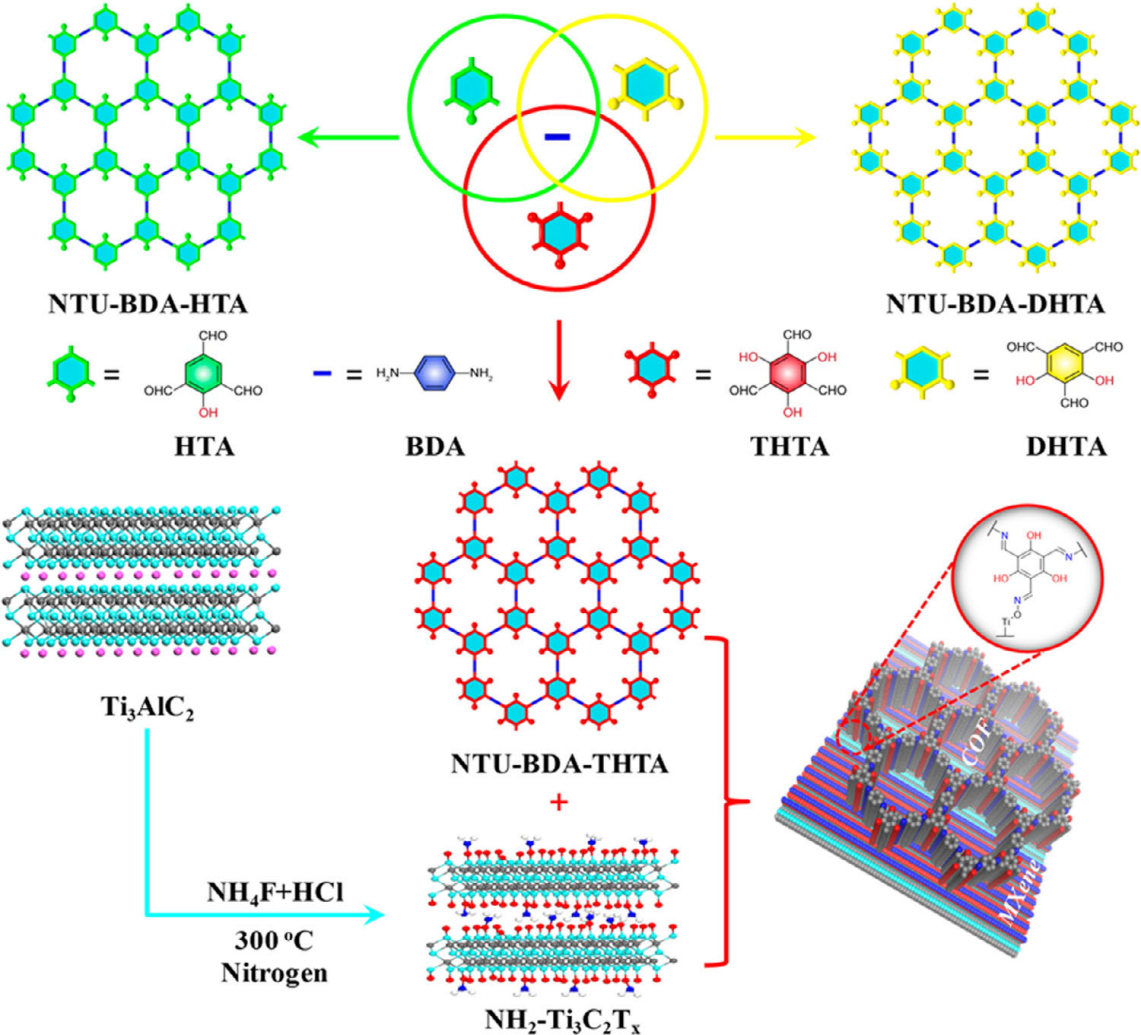
Synthesis of β‐ketoenamine‐kinked covalent organic frameworks (COFs) and hybridization with NH_2_‐MXenes. Adapted with permission.^[^ ^234^ ^]^ Copyright 2020, American Chemical Society.

COFs have received considerable attention in catalysis.^[^ ^270^ ^]^ However, there remains a big challenge in improving the photochemical activity of COFs. Zhao et al. changed the ratio of β‐ketoenamine as well as imine, and successfully prepared diverse COFs for photocatalytic hydrogen production.^[^ ^234^ ^]^ Inspired by the appropriate HOMO energy of COFs, nonquenched excited state, and planar and moderately ordered structure, covalent coupling with NH_2_‐Ti_3_C_2_T*_x_* enables the formation of COF/NH_2_‐Ti_3_C_2_T*_x_* composites. The photocatalytic experiment and theoretical analysis showed that the more β‐ketoenamine groups, the better the photocatalytic performance. (Figure 18). The synergistic effect of the COF/NH_2_‐Ti_3_C_2_T*_x_* heterostructure can not only achieves efficient separation as well as the transfer of photogenerated charge carriers, but also accelerates surface proton reduction.

## 3 CONCLUSION AND PERSPECTIVE

Indeed, COFs‐based composites have certain advantages that distinguish them from other composites applied in various applications. Compared with other materials, it can be ingeniously designed and constructed. In addition, by choosing appropriate building blocks and linking motifs, materials with tunable pore sizes, defined chemical structures, and low‐density can be obtained, which are not commonly found in other typical composite materials.

The structures and properties of the parent COFs are the main considerations when designing composites. Although multiple COFs‐based composites have been reported, some fundamental problems also need to be solved urgently: (1) The organic monomer used to synthesize COFs is relatively expensive, which makes the synthesis cost of COFs‐based composites high. (2) During the synthesis process, a series of preparation approaches have been established (solvothermal synthesis) for the preparation of diverse COFs and COFs‐based composites with high crystallinity and harsh reaction conditions (inert atmosphere, high pressure/temperature, and sealed Pyrex tubes), which makes it rather difficult to control the morphology and size of COFs‐based composites, let alone large‐scale production and practical applications. (3) In practical applications, many properties of COFs‐based composites are usually superior to those of single materials. However, in terms of hydrophilic and hydrophobic properties, most of these materials are poor in hydrophilicity, and the robustness and mechanism of the composites remain to be investigated. (4) The long‐term biotoxicity of COFs‐based composites in vivo has not been fully investigated. Besides, it has been shown that the current studies mainly focus on a few “classic” COFs. More alternative chemically and thermally stable porous COFs are urgently required.

Of course, green, economical synthetic strategies as well as mild reaction conditions are still the main direction of research efforts. Meanwhile, more assisted techniques such as computer simulation, machine learning are expected to better provide theoretical data for the design, characterization, and application of COFs‐based composites. It is still essential to further study the mechanism between COFs and the corresponding functional materials in the reaction processes to obtain composites with better and more stable performance by combining specific materials with specific problems. In addition, it is also necessary to find new potential materials to construct COFs‐based composites with more practicability, biocompatibility, and low‐toxicity. Finally, we believe that COFs‐based composites will certainly continue to emerge, and the different properties they bring will be more widely used in various research fields.

## CONFLICT OF INTEREST STATEMENT

The authors declare no conflicts of interest.
